# *Olsenella scatoligenes*-derived skatole promotes smooth muscle cell proliferation and migration to aggravate atherosclerosis

**DOI:** 10.1093/ismejo/wraf238

**Published:** 2025-10-23

**Authors:** Yawen Zhao, Jiarui Chen, Shanshan Zhu, Yingxi Xu, Jiangyuan Zhu, Jialu Yang, Weibin Zhou, Ying Yang, Maohuan Lin, Qian Chen, Min Xia, Yangxin Chen, Yan Liu

**Affiliations:** Guangdong Provincial Key Laboratory of Food, Nutrition and Health, and Department of Nutrition, School of Public Health Sun Yat-sen University, Guangzhou, P.R. China; Guangdong Provincial Key Laboratory of Food, Nutrition and Health, and Department of Nutrition, School of Public Health Sun Yat-sen University, Guangzhou, P.R. China; Guangdong Provincial Key Laboratory of Food, Nutrition and Health, and Department of Nutrition, School of Public Health Sun Yat-sen University, Guangzhou, P.R. China; Guangdong Provincial Key Laboratory of Food, Nutrition and Health, and Department of Statistics and Epidemiology, School of Public Health Sun Yat-sen University, Guangzhou, P.R. China; Guangdong Provincial Key Laboratory of Food, Nutrition and Health, and Department of Nutrition, School of Public Health Sun Yat-sen University, Guangzhou, P.R. China; Guangdong Provincial Key Laboratory of Food, Nutrition and Health, and Department of Nutrition, School of Public Health Sun Yat-sen University, Guangzhou, P.R. China; Department of Cardiology, Sun Yat-sen Memorial Hospital, Sun Yat-sen University, Guangzhou, P.R. China; Guangdong Province Key Laboratory of Arrhythmia and Electrophysiology, Sun Yat-sen Memorial Hospital, Sun Yat-sen University, Guangzhou, P.R. China; Department of Cardiology, Sun Yat-sen Memorial Hospital, Sun Yat-sen University, Guangzhou, P.R. China; Guangdong Province Key Laboratory of Arrhythmia and Electrophysiology, Sun Yat-sen Memorial Hospital, Sun Yat-sen University, Guangzhou, P.R. China; Department of Cardiology, Sun Yat-sen Memorial Hospital, Sun Yat-sen University, Guangzhou, P.R. China; Guangdong Province Key Laboratory of Arrhythmia and Electrophysiology, Sun Yat-sen Memorial Hospital, Sun Yat-sen University, Guangzhou, P.R. China; Department of Cardiology, Sun Yat-sen Memorial Hospital, Sun Yat-sen University, Guangzhou, P.R. China; Guangdong Province Key Laboratory of Arrhythmia and Electrophysiology, Sun Yat-sen Memorial Hospital, Sun Yat-sen University, Guangzhou, P.R. China; Guangdong Provincial Key Laboratory of Food, Nutrition and Health, and Department of Nutrition, School of Public Health Sun Yat-sen University, Guangzhou, P.R. China; Department of Cardiology, Sun Yat-sen Memorial Hospital, Sun Yat-sen University, Guangzhou, P.R. China; Guangdong Province Key Laboratory of Arrhythmia and Electrophysiology, Sun Yat-sen Memorial Hospital, Sun Yat-sen University, Guangzhou, P.R. China; Guangdong Provincial Key Laboratory of Food, Nutrition and Health, and Department of Nutrition, School of Public Health Sun Yat-sen University, Guangzhou, P.R. China

**Keywords:** *Olsenella scatoligenes*, skatole, atherosclerosis

## Abstract

Coronary artery disease (CAD) remains the leading cause of mortality and morbidity globally. The gut microbiota has been implicated in the development of CAD through unclear mechanisms. Here, we demonstrate that the abundance and interspecies interactions of *Olsenella scatoligenes* are 4.7- and 1.6-fold higher in patients with CAD, respectively, and positively associated with disease severity. Furthermore, integrative metagenomic and metabolomic analyses identify skatole as the key microbial effector mediating the pro-atherogenic effect of *O. scatoligenes*. Consistently, supplementation with *O. scatoligenes* or skatole results in 1.26- and 1.23-fold increases in aortic plaque area, respectively, by promoting vascular smooth muscle cell proliferation and migration to the intima. Mechanistically, *O. scatoligenes*-derived skatole facilitates nuclear translocation of the aryl hydrocarbon receptor and enhances its binding to the promoter region of *calponin 1*. Silencing either *aryl hydrocarbon receptor* or *calponin 1* attenuates ~40% of the vascular smooth muscle cell proliferation and migration induced by skatole. Collectively, our study identifies increased skatole production as the principal microbial effector linking *O. scatoligenes* to aggravated atherosclerosis through activation of the aryl hydrocarbon receptor-calponin 1 axis and underscores the therapeutic potential of targeting skatole production for the management of CAD.

## Introduction

Coronary artery disease (CAD), resulting from atherosclerotic lesions in coronary arteries, remains the leading cause of mortality and a major contributor to global disability [1, 2]. As a principal structural component of the vessel wall, vascular smooth muscle cells (VSMCs) play an essential role in maintaining normal vascular functions, including vasodilation and contraction. During atherosclerosis, VSMCs proliferate, migrate into the intimal layer of the arterial wall [3–6], and undergo dedifferentiation from a quiescent, contractile state to a proliferative phenotype [7, 8]. Lineage tracing studies have further demonstrated that a substantial proportion of cells within atherosclerotic lesions originate from differentiated medial VSMCs [7–9]. As the disease advances, these dedifferentiated VSMCs secrete chemokines and pro-inflammatory cytokines and disrupt collagen and fibronectin assembly, thereby promoting plaque progression and rupture [7, 8, 10]. Thus, targeting VSMCs represents a promising therapeutic strategy for the management of CAD.

Mounting evidence indicates that gut microbiota dysbiosis contributes to the pathogenesis of CAD through multiple mechanisms, including low-grade endotoxemia, altered short-chain fatty acid production, and bile acid dysregulation [11, 12]. The microbiome of patients with atherosclerotic cardiovascular diseases exhibits lower diversity but higher abundances of *Streptococcus* species and *Enterobacteriaceae* family, as well as unfavorable metabolic capacities, such as enhanced lipopolysaccharide biosynthesis and increased protein and tryptophan metabolism, compared to healthy controls [13]. Several indole derivatives, including indole-3-propionic acid and indole-3-carboxaldehyde, have been implicated in the regulation of endothelial function and vascular inflammation [14, 15]. Moreover, the genus *Olsenella* has been found to be enriched in individuals with metabolic disorders and positively associated with interferon-α, a pro-inflammatory cytokine relevant to atherosclerosis. Germ-free mice receiving fecal microbiota transplantation from patients with CAD exhibited adverse lipid profiles and accelerated vascular aging [16], whereas, supplementation with probiotics such as *Akkermansia muciniphila*, alleviates atherosclerosis progression by suppressing local inflammation [11].

Despite findings in murine models and western populations, the major bacterium and molecular mechanisms whereby altered gut microbiota promotes CAD in Chinese populations remain to be elucidated. To address this gap, we performed an integrative metagenomic and metabolomic analysis in patients with angiographically confirmed CAD and non-CAD controls, and *Olsenella scatoligenes* (*O. scatoligenes*) was found to be a major bacterium in the progression of CAD. Furthermore, a series of *in vivo* and *in vitro* experiments were conducted to elucidate the underlying mechanism linking *O. scatoligenes* to exacerbated atherosclerosis.

## Materials and methods

### Study population

Participants in both discovery (*n* = 149) and validation (*n* = 179) cohorts were recruited at Sun Yat-sen Memorial Hospital, Sun Yat-sen University (Guangzhou, China). The study protocol was approved by the Ethics Committee of the School of Public Health, Sun Yat-Sen University (2018-021), and was conducted in accordance with the Declaration of Helsinki. Written informed consent was obtained from all individuals prior to enrollment.

### *Olsenella scatoligenes* culture

*Olsenella scatoligenes* was obtained from the DSMZ (German Collection of Microorganisms and Cell Cultures GmbH, DSM28304) and cultured at 37°C under anoxic conditions in peptone-yeast paste-glucose medium. Cells were harvested by centrifugation at 3000 rpm for 5 min at room temperature, and resuspended in sterile reduced phosphate buffered saline (PBS) containing 10% glycerin. The bacterial suspension was adjusted to a final density of 5 × 10^8^ colony-forming units (CFUs) per 200 μl and freshly prepared prior to gavage. For pasteurization, suspensions were heated at 70°C for 30 min in a calibrated water bath (gentle inversion every 10 min) and then immediately chilled on ice [17]. The strain identity and purity were confirmed by sequencing with universal primers 27 F/1492 R on the Sanger platform, and alignment with reference strains using BLAST against the NCBI 16S ribosomal RNA (16S rRNA) genes sequence database prior to use.

### Animal model

All procedures were reviewed and approved by the Animal Care and Utilization Committee of Sun Yat-sen University (2018-009). Eight-week-old male apolipoprotein E-deficient (apoE^−^/^−^; C57BL/6 J background) mice were obtained from GemPharmatech Technology (Guangzhou, China). Mice were randomly assigned to experimental groups via block randomization with SPSS software (v25.0; IBM, Armonk, NY, USA) and housed under a specific pathogen-free environment at 23 ± 2°C, with a 12-h light–dark cycle and free access to food and water.

ApoE^−^/^−^ mice were maintained on a high-fat, high-cholesterol (HFHC) diet (Medicience, Professionals for Lab Animal Diets, Nanjing, China) for 12 weeks to establish atherosclerosis. Live *O. scatoligenes* or pasteurized *O. scatoligenes* at a dose of 5 × 10^8^ CFU per 200 μl, or skatole at a dose of 50 mg/kg, were gavaged on a daily basis for 12 weeks. Reduced PBS with 10% glycerin was used as a vehicle control for *O. scatoligenes* intervention, whereas 0.3% carboxymethylcellulose sodium (CMC-Na) was used as a solvent control for skatole intervention.

To suppress *CNN1* expression in VSMCs, an adeno-associated virus 9 (AAV9) system driven by the SM22α promoter was employed. AAV9-SM22α-Cnn1 and AAV9- SM22α-NC (negative control) were purchased from Genomeditech Co. Ltd (Shanghai, China). After receiving a single tail vein injection of 1 × 10^11^ viral particles (in 100 μl sterile PBS), apoE^−^/^−^ mice fed with an HFHC diet were gavaged with 0.3% CMC-Na or skatole (50 mg/kg/day) for 12 weeks before analysis.

After 12-week intervention, mice were anesthetized by intraperitoneal injection of pentobarbital sodium (50 mg/kg body weight). Once a sufficient depth of anesthesia was confirmed by the absence of reflex responses, blood was collected via retro-orbital sinus puncture using a heparinized capillary tube. Following blood collection, the animals were euthanized by carbon dioxide (CO₂) inhalation in accordance with current National Institutes of Health Guide for the Care and Use of Laboratory Animals (NIH guidelines). Tissues were then harvested for further analysis.

### Cell culture

Human aortic smooth muscle cells (HASMCs, CRL-1999) and HEK293T cells (CRL-3216) were purchased from the American Type Culture Collection. Both cell lines were cultured in high-glucose Dulbecco's Modified Eagle Medium (DMEM, Gibco, Grand Island, CA, USA) supplemented with 10% fetal bovine serum (FBS, Gibco, Australia), 100 U/ml penicillin (Gibco, Grand Island, CA, USA), and 100 μg/ml streptomycin (Gibco, Grand Island, CA, USA). Cells were maintained at 37°C in a humidified incubator with 5% CO_2_.

### Statistical analysis

For human studies, statistical analyses were performed using R software (version 4.0.3), unless otherwise specified. Data normality was evaluated using the Shapiro–Wilk test and Q–Q plots. Normally distributed variables were expressed as mean ± SD and compared by Student’s *t*-test, whereas non-normally distributed variables were summarized as median (interquartile range) and analyzed with the Wilcoxon rank-sum test. Categorical variables were compared using the chi-squared test or Fisher’s exact test, as appropriate. To account for multiple testing, adjusted *P* values were calculated using the Benjamini–Hochberg false discovery rate.

For animal studies, sample size was determined based on prior studies and no statistical method was used to predetermine the sample size. Experiments were carried out with technical replicates, and the mean value of technical replicates was used for each biological replicate. Statistical analyses were conducted using GraphPad Prism 9.5, and data were presented as mean ± SEM. Comparisons between two groups were conducted using Student’s *t*-test, comparisons among three groups were performed using one-way analysis of variance (ANOVA) followed by Tukey’s test and two-way ANOVA for four groups.

Further details regarding study participants, animal experiments, *in vitro* assays, and multi-omics analyses were provided in the Supplementary Materials.

## Results

### *Olsenella scatoligenes* is enriched in patients with CAD

**Figure 1:**
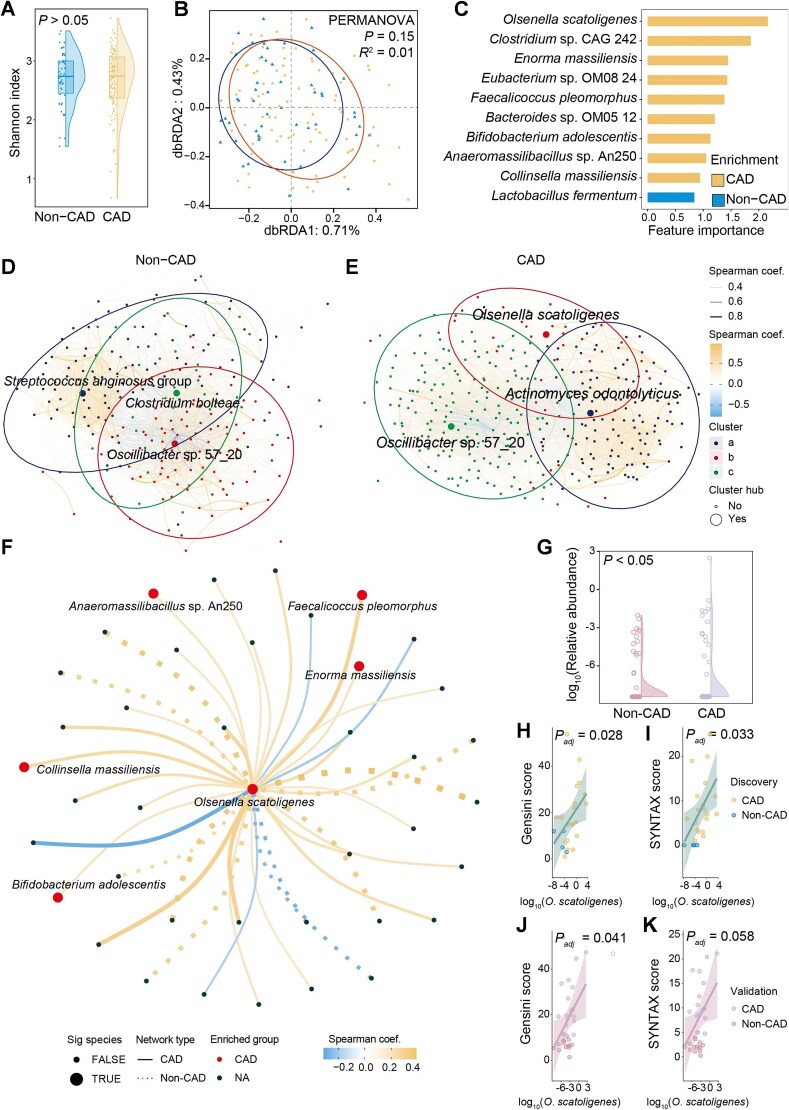
Increased *O. scatoligenes* is associated with the progression of atherosclerotic plaque. (A) Alpha-diversity (Shannon index) and (B) beta diversity of gut microbiota in subjects with or without CAD. (C) Species independently associated with CAD (*P_adj_* < 0.2). Co-abundance network of the gut microbiota in (D) non-CAD controls and (E) patients with CAD. The node was colored according to their affiliated clusters and only the names of hub species were shown. (F) Correlations between *O. scatoligenes* and other species in the co-abundance network of CAD and non-CAD controls. (G) The distribution of *O. scatoligenes* in subjects with or without CAD in an independent validation cohort. Association of *O. scatoligenes* with (H and J) Gensini score and (I and K) SYNTAX score in discovery cohort and validation cohort, respectively. *P* values were derived from multivariate linear regression adjusted for age, sex, BMI, and medication use.

To better understand the role of gut microbiota in CAD, metagenomic sequencing was performed on fecal samples from 99 patients with CAD and 50 non-CAD controls. Compared with controls, patients with CAD were more likely to be male and to have hypertension, adverse lipid profiles, and higher statin use (Table S1). Although no significant difference in microbial diversity was observed (Fig. 1A and B), after adjustment for age, sex, body mass index (BMI), and medication use, 10 species remained significantly associated with CAD. Among them, *O. scatoligenes*, *Clostridium* sp. CAG 24*2* and *Enorma massiliensis* were highly enriched, whereas *Lactobacillus fermentum*, a probiotic known to enhance immune responses [18], was substantially depleted in patients with CAD (Fig. 1C). Given that gut microbes form complex interactive communities rather than existing independently [19], we further examined microbial co-occurrence networks in CAD and non-CAD individuals. Overall, network node degree was significantly higher (Fig. S1A) whereas closeness was significantly lower (Fig. S1B) in the network of patients with CAD, suggesting a hub-and-spoke microbial community structure (Fig. S1). When further clustered into different subgroups, *Clostridium bolteae* and *Streptococcus anginosus group* acted as hub species unique to non-CAD networks, whereas, *Actinomyces odontolyticus* and *O. scatoligenes* were identified as potential hub species specifically in the microbiome of patients with CAD (Fig. 1D and E). *Olsenella scatoligenes* was the only hub species that was also significantly enriched in patients with CAD. Significant connections with *O. scatoligenes* increased from 11 in the community of non-CAD controls to 29 in the microbiome of patients with CAD, among which strong positive interactions were found between *O. scatoligenes* and multiple species enriched in patients with CAD, such as *Collinsella massiliensis*, *Anaeromassilibacillus* sp*.* An250, *Bifidobacterium adolescentis*, *E. massiliensis*, and *Faecalicoccus pleomorphus* (Fig. 1F), underscoring its central role in the CAD-associated microbiome. In the validation cohort, despite milder disease severity (Table S2), the median abundance of *O. scatoligenes* was also found to be 106% higher in patients diagnosed with CAD compared to non-CAD controls. (Fig. 1G). After adjustment for age, sex, BMI, and medication use, *O. scatoligenes* showed weak associations with traditional CAD risk factors (Fig. S2), but strong positive correlations with both the Gensini score (Fig. 1H and J) and the SYNTAX score (Fig. 1I and K), two widely used indicators of CAD severity, in both discovery and validation cohorts. Collectively, the above findings indicated that *O. scatoligenes* might be involved in the progression of CAD independent of traditional risk factors.

### *Olsenella scatoligenes* accelerates the progression of atherosclerosis

**Figure 2:**
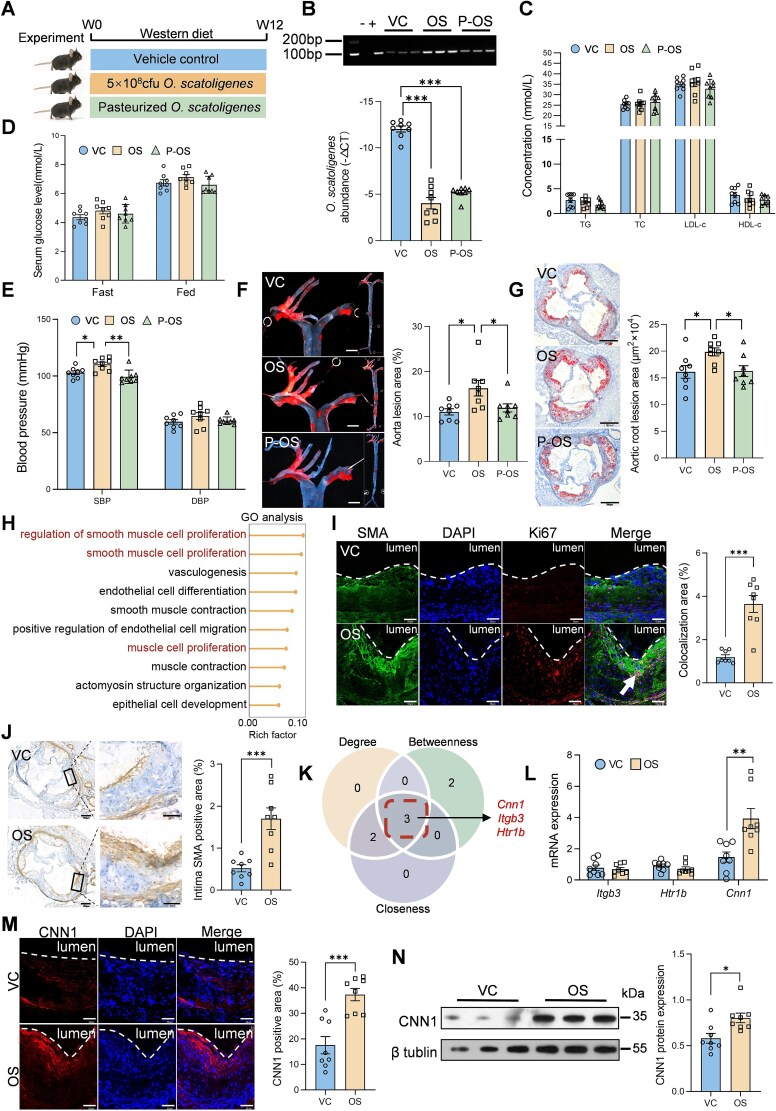
*Olsenella scatoligenes* supplementation accelerates atherosclerosis in mice. ApoE^−/−^ mice were gavaged with *O. scatoligenes* (OS), vehicle control (VC) or pasteurized *O. scatoligenes* (P-OS) for 12 weeks before analysis. (A) Schematic diagram of the study design. (B) Quantification of *O. scatoligenes* in feces. (C) Serum lipid, (D) glucose, and (E) blood pressure. (F and G) Representative image and quantification of Oil Red O staining in (F) aorta (scale bar, 100 px) and (G) cross-sections of aortic root (scale bar, 200 μm). (H) Gene ontology enrichment analysis of differentially expressed genes in the aorta of mice treated with *O. scatoligenes* or vehicle control. Representative image and quantification of (I) the proliferation of VSMC (scale bar, 50 μm) and (J) VSMC in the intima of the aorta (scale bar, 200 and 50 μm, respectively). (K) Venn chart showing the intersection of genes with significant node degree, node betweenness, and node closeness. (L) Relative expression of potential target genes by RT-qPCR analysis in the aorta. Expression of CNN1 in the aorta determined by (M) immunofluorescent staining (scale bar, 50 μm) and (N) western blot analysis. *P* values were determined by one-way ANOVA followed by Tukey’s test for B–G and Student’s *t* test for I and J and L–N. Data were represented as mean ± SEM (*n* = 8 mice per group), ^*^P < .05, ^**^P < .01, and ^***^P < .001.

To further validate the pro-atherogenic effect of *O. scatoligenes*, apoE^−^/^−^ mice were orally gavaged with live *O. scatoligenes*, pasteurized *O. scatoligenes*, or vehicle control for 12 weeks (Fig. 2A). Following gavage, the relative abundance of *O. scatoligenes* in fecal samples increased ~3-fold (Fig. 2B), confirming successful colonization. Although no significant differences in serum lipids (Fig. 2C) or glucose levels (Fig. 2D) were observed among the three groups, systolic blood pressure (SBP), but not diastolic blood pressure (DBP), was significantly elevated in mice receiving live *O. scatoligenes* (Fig. 2E). Consistently, plaque area in mice gavaged with live *O. scatoligenes* was 1.26- and 1.23-fold greater than that of the vehicle-treated group in the entire aorta (Fig. 2F) and aortic root regions (Fig. 2G), respectively. In contrast, pasteurized *O. scatoligenes* did not elicit such a pro-atherogenic effect (Fig. 2E–G), suggesting that translocation of metabolites derived from or induced by *O. scatoligenes* may be responsible for its pro-atherogenic effect.

To explore the molecular mechanisms by which *O. scatoligenes* exacerbates atherosclerotic plaque formation, the aorta was subjected to RNA sequencing analysis. Compared to mice treated with vehicle control, 842 genes were differentially expressed in those receiving live *O. scatoligenes* (Fig. S3A). Gene Ontology enrichment analysis revealed that pathways involved in VSMC proliferation and migration were substantially enhanced in mice challenged with live *O. scatoligenes* (Fig. 2H). In line with the functional inference, a 3-fold increase in the proliferation of VSMCs was found in mice gavaged with live *O. scatoligenes* (Fig. 2I), accompanied by a significantly higher accumulation of VSMCs in the intima (Fig. 2J), indicating a transition to dedifferentiated VSMCs. No significant difference in macrophage infiltration, inflammation, or collagen deposition (Fig. S4) was found between mice treated with vehicle control and *O. scatoligenes*, further supporting the specific involvement of *O. scatoligenes* in promoting VSMC proliferation and migration. Among the co-expression network of 25 significant genes involved in VSMC proliferation (Fig. S3B), 5-hydroxytryptamine receptor 1B, calponin 1 (*Cnn1*), and integrin subunit beta 3 were identified as potential keystone genes based on degree, betweenness, and closeness centrality (Fig. S3C–E and Fig. 2K). Of these, *Cnn1* remained significantly upregulated in RT-qPCR validation (Fig. 2L). Consistently, CNN1 protein expression was more than doubled in the aortic intima of mice treated with live *O. scatoligenes* (Fig. 2M and N), suggesting that CNN1 may be a key downstream target of *O. scatoligenes*. Collectively, these results demonstrate that *O. scatoligenes* exacerbates atherosclerosis by promoting VSMC proliferation and migration.

### Skatole is the key microbial effector linking *O. scatoligenes* to atherosclerosis

**Figure 3:**
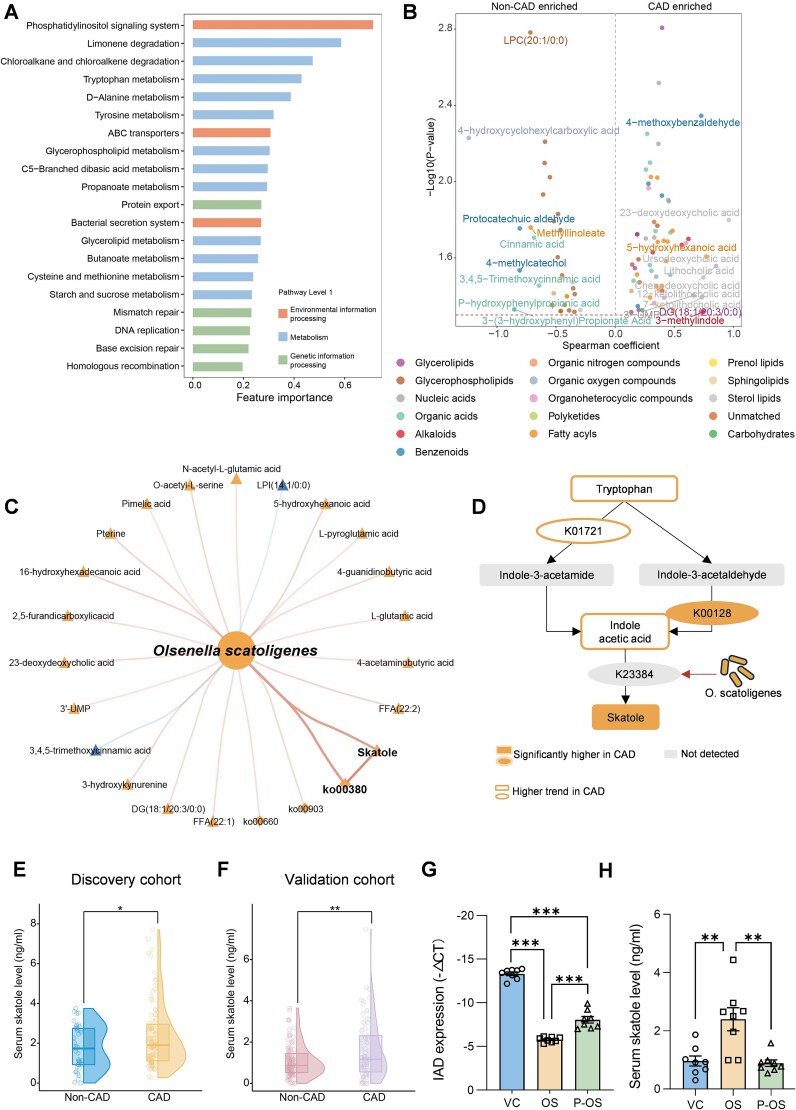
Microbial production of skatole is upregulated in patients with CAD. (A) Microbial functions independently associated with CAD. (B) Volcano plot showing the differentially enriched metabolites between CAD patients and non-CAD controls. For clarity, only the names of metabolites with the top 20 feature importance were shown. The colors indicated the classification of metabolites. (C) Correlation network among the abundance of *O. scatoligenes*, microbial functions, and metabolites. (D) Schematic diagram of the microbial production of skatole. Plasma levels of skatole in (E) discovery (*n* = 131) and (F) validation cohort (*n* = 176). (G) Indoleacetate decarboxylase expression in fecal samples. (H) Serum levels of skatole in mice gavaged with *O. scatoligenes* or vehicle control. *P* was determined by multivariate regression adjusted for sex, age, BMI, and medication use for E and F, and one-way ANOVA followed by Tukey’s test for G and H (*n* = 8 mice per group), ^*^P < .05, ^**^P < .01, and ^***^P < .001.

To elucidate how *O. scatoligenes* influences CAD, we integrated metagenomic and fecal metabolomic analyses. After adjustment for age, sex, BMI, and medication use, 20 microbial functional capacities, mainly involving amino acid metabolism, carbohydrate and lipid metabolism, were significantly associated with CAD (Fig. 3A). Similarly, 97 fecal metabolites, predominantly glycerophospholipids, fatty acyls, and organic acids, were independently linked to CAD progression, after correcting for potential confounders (Fig. 3B). Overall, the pattern of microbial metabolites corresponded closely with functional shifts in microbial pathways. For instance, increased levels of L-glutamic acid and L-pyroglutamic acid in CAD patients were consistent with enhanced tryptophan metabolism. Likewise, several carbohydrate-related metabolites, including pimelic acid, azelaic acid, and carnitine, were elevated in CAD patients compared to non-CAD controls. In addition, aligned with enhanced lipid metabolic activities, several lipids, such as lysophosphatidic acid 0:0/18:0, multiple diacylglycerols, and phosphatidylserine species, were found to be significantly higher in CAD patients. Among these altered pathways and metabolites, only three microbial capacities (i.e. limonene degradation, tryptophan metabolism, and C5-branched dibasic acid metabolism) showed positive correlations with the abundance of *O. scatoligenes*, whereas 18 metabolites, such as skatole, L-glutamic acid, and L-pyroglutamic acid, were positively correlated with *O. scatoligenes* (Fig. 3C).

The strong positive correlations among *O. scatoligenes*, tryptophan metabolism, and skatole were further supported by biological evidence. Among the 17 fecal metabolites and 12 microbial genes associated with tryptophan metabolism detected in our cohort, only skatole and its upstream enzyme aldehyde dehydrogenase (ALDH, K00128) showed consistent positive associations with both CAD status and the abundance of *O. scatoligenes*, whereas other tryptophan-related metabolites, such as 2-aminophenol and 2-aminobenzoic acid, displayed discordant associations (Fig. S5). Specifically, tryptophan was converted to indole-3-acetic acid via nitrile hydratase subunit alpha (*nthA*, K01721) and aldehyde dehydrogenase (ALDH, K00128), which was then transformed into skatole by indoleacetate decarboxylase (*iad*, K23384, Fig. 3D). As a key enzyme catalyzing the terminal step of skatole biosynthesis, the *iad* gene was annotated in the genome of *O. scatoligenes* (Fig. S6A). This functional inference was further corroborated by the exclusive detection of skatole in the culture medium of *O. scatoligenes*, instead of *Alistipes indistinctus* which lacked the gene encoding *iad* (Fig. S6B and C).

Although fecal metabolism provided a better functional readout of microbial activity [20], we further quantified circulating skatole to assess how *O. scatoligenes* affected the distal aorta. Plasma skatole concentrations were 1.3- and 1.5-fold higher in patients with CAD, compared to non-CAD controls in both discovery (Fig. 3E) and validation cohorts (Fig. 3F), after correcting for major confounders. Circulating skatole was positively correlated with atherosclerotic burden, as evidenced by positive correlations with both Gensini score (Discovery: *ρ* = 0.20, *P* = .022; Validation: *ρ* = 0.19, *P* = .012) and SYNTAX score (Discovery: *ρ* = 0.18, *P* = .04; Validation: *ρ* = 0.21, *P* = .006, Fig. S7). Consistently, colonization with live *O. scatoligenes* in mice led to elevated fecal *iad* expression (Fig. 3G) and skatole in the circulation (Fig. 3H), which was absent in mice treated with pasteurized *O. scatoligenes*. Together, these findings identify skatole as the microbial effector linking *O. scatoligenes* to exacerbated atherosclerosis.

### Skatole aggravates proliferation and migration of VSMCs via activation of CNN1

**Figure 4:**
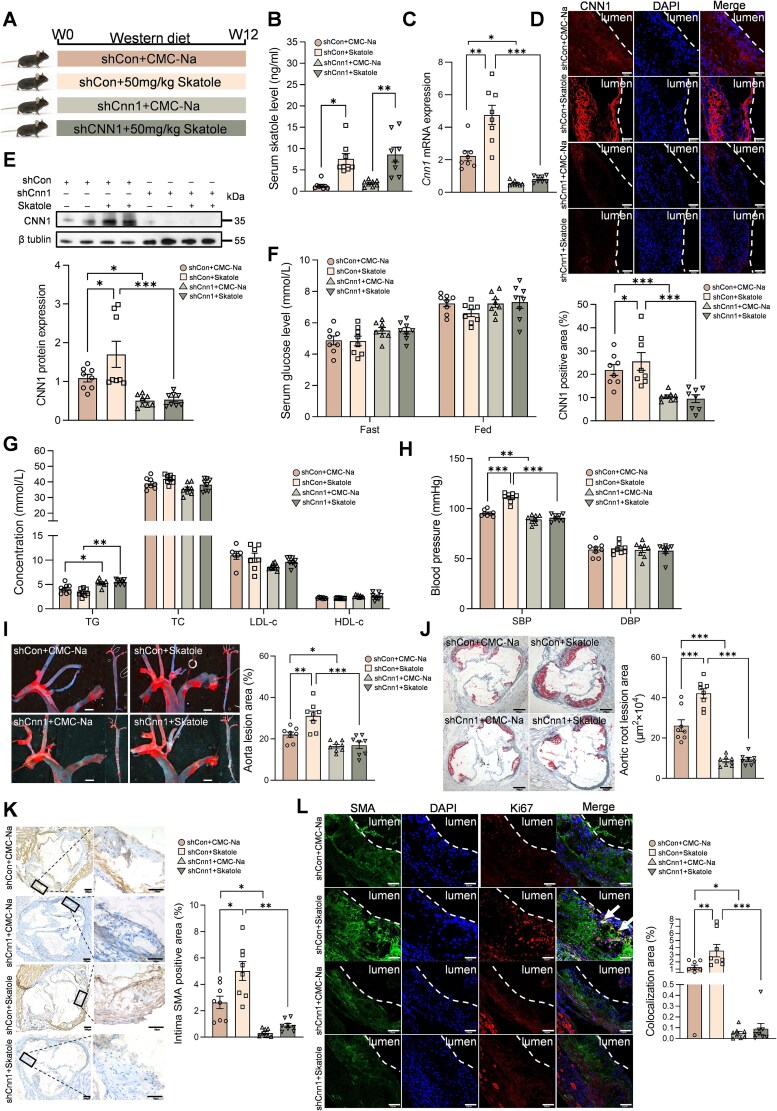
Administration of skatole exacerbates atherosclerotic plage formation in mice. ApoE^−/−^ mice receiving either control AAV9 vector (shCon) or AAV9 targeting Cnn1 (shCnn1) through tail vein were gavaged with 50 mg/kg skatole or CMC-Na for 12 weeks before analysis. (A) Schematic diagram of the study design. (B) Quantification of serum skatole. Expression of *Cnn1* at (C) mRNA level and protein level determined by (D) immunofluorescent staining (scale bar, 50 μm) and (E) western blot analysis, respectively. (F) Serum glucose, (G) lipid, and (H) blood pressure. (I and J) Representative image and quantification of Oil Red O staining in (I) aorta (scale bar, 100 px) and (J) cross-sections of aortic root (scale bar, 200 μm). Representative image and quantification of (K) intima VSMC (scale bar, 200 and 50 μm, respectively). (L) The proliferation of VSMC in the aorta (scale bar, 50 μm). *P* values were determined by two-way ANOVA for B–L. Data were represented as mean ± SEM (*n* = 8 mice per group). ^*^P < .05, ^**^P < .01, and ^***^P < .001.

**Figure 5:**
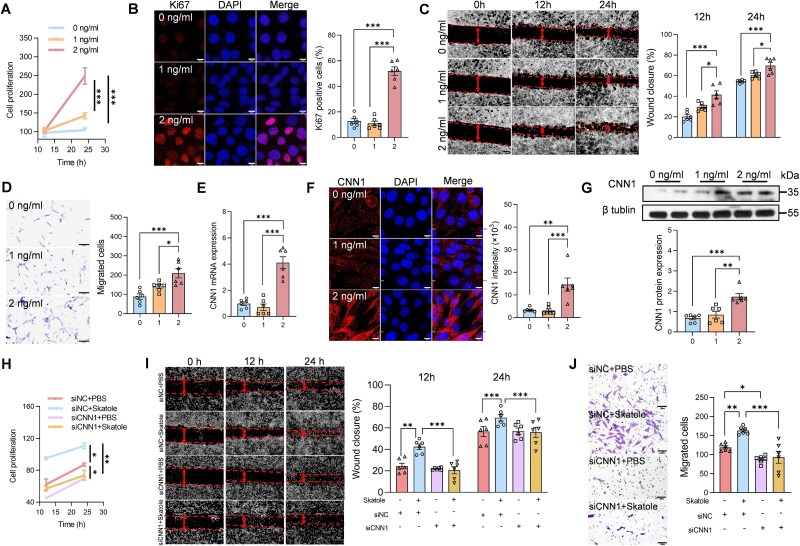
Skatole promotes the proliferation and migration of VSMC. (A–G) HASMCs were treated with skatole at indicated doses for 12 or 24 h before analysis. (A) Proliferation of HASMCs. (B) Representative image and quantification of Ki67^+^ cells (scale bar, 10 μm). Representative image and quantification of cell migration determined by (C) wound-healing assay (scale bar, 20 μm) and (D) transwell assay (scale bar, 100 μm). Expression of CNN1 at (E) mRNA level, and protein level determined by (F) immunofluorescent staining (scale bar, 10 μm) and (G) western blot analysis, respectively. (H–J) HASMCs were transfected with siRNA targeting *CNN1* for 24 h, followed by skatole treatment for another 24 h before analysis. (H) Proliferation of HASMCs. Representative image and quantification of cell migration determined by (I) wound-healing assay (scale bar, 20 μm) and (J) transwell assay (scale bar, 100 μm). *P* values were determined by one-way ANOVA followed by Tukey’s test. Data were represented as mean ± SEM (*n* = 6 independent experiments). ^*^P < .05, ^**^P < .01, and ^***^P < .001.

We examined whether *O. scatoligenes*-derived skatole promotes atherosclerosis in a CNN1-dependent manner. ApoE^−^/^−^ mice transduced with either control AAV9 vector (shCon) or AAV9 targeting *Cnn1* (shCnn1) were gavaged with skatole or vehicle control (CMC-Na) for 12 weeks (Fig. 4A and Fig. S8A and B). Compared with vehicle control, skatole gavage resulted in a 3-fold increase in circulating skatole, reaching levels comparable to those observed in patients with severe CAD (Fig. 4B). Moreover, skatole treatment induced a 2.1-fold increase in CNN1 expression in the aorta at both mRNA (Fig. 4C) and protein levels (Fig. 4D and E). Similar to the observation in mice gavaged with *O. scatoligenes*, no significant differences in glucose or lipid metabolism (Fig. 4F and G), macrophage activation, inflammatory cytokine expression, and collagen deposition (Fig. S8C–E) were found in mice exposed to skatole. However, mice treated with skatole exhibited a marked elevation in SBP (Fig. 4H) and significantly larger atherosclerotic lesions (Fig. 4I and J), accompanied by ~2-fold increases in the proliferation and migration of VSMCs to the intimal layer of the arterial wall (Fig. 4K and L). In contrast, suppression of CNN1 expression by ~75% in the aorta (Fig. 4C–E and Fig. S8A and B) largely abolished the skatole-induced elevation of SBP, lesion burden, and VSMC proliferation and migration (Fig. 4H–L). Similarly, exposure of HASMCs to skatole at concentrations mimicking healthy (0 ng/ml), non-CAD (1 ng/ml) and CAD (2 ng/ml) levels led to enhanced proliferation and migration only at the higher concentration (Fig. 5A–D), accompanied by increased CNN1 expression (Fig. 5E–G). Silencing of *CNN1* (Fig. S9) substantially reduced the skatole-induced proliferation and migration of HASMCs (Figs. 5H–J). Collectively, these results demonstrate that CNN1 is required for skatole-induced proliferation and migration of VSMCs.

### AHR is required for CNN1-mediated proliferation and migration by skatole

**Figure 6:**
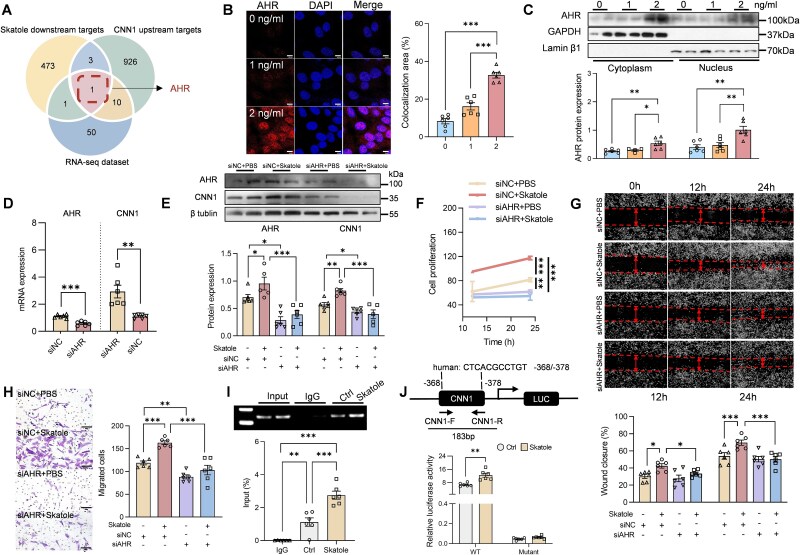
AHR-CNN1 axis is required for the proliferation and migration of HASMCs induced by skatole. (A) Overlap of potential downstream transcription factors of skatole, *CNN1* upstream regulators, and significantly altered transcription factors in RNA-sequencing data. (B and C, I and J) HASMCs were treated with skatole at indicated doses for 24 h before analysis. (B) Nucleus translocation of AHR (scale bar, 10 μm). (C) Expression of AHR in the cytoplasm and nucleus. (D–H) HASMCs were transfected with siRNA targeting *AHR* for 24 h, followed by skatole treatment for another 24 h before analysis. Expression of AHR and CNN1 at both (D) mRNA and (E) protein levels. (F) Proliferation of HASMCs. Representative image and quantification of cell migration determined by (G) wound-healing assay (scale bar, 20 μm) and (H) transwell assay (scale bar, 100 μm). (I) Binding capacity of AHR to *CNN1* promoter upon skatole treatment. (J) Cells were transfected with empty vector or the *CNN1* promoter plasmid with or without a mutated AHR-binding site and then treated with skatole before measuring *CNN1* promoter luciferase activity. *P* values were determined by one-way ANOVA followed by Tukey’s test. Data were represented as mean ± SEM (*n* = 6 independent experiments). ^*^P < .05, ^**^P < .01, and ^***^P < .001.

To further elucidate the mechanism by which skatole induces *CNN1* transcription, we integrated *in silico* predictions with RNA-sequencing data. Among potential downstream targets of skatole, transcription factors likely to regulate *CNN1*, and differentially expressed transcription factors detected by RNA sequencing in the aorta of mice treated with *O. scatoligenes* or vehicle, aryl hydrocarbon receptor (AHR) was identified as a candidate regulator (Fig. 6A). Consistent with this observation, treatment with skatole at concentrations comparable to those detected in CAD patients promoted nuclear translocation of AHR, resulting in a 2.5-fold increase in nuclear AHR levels (Fig. S10A and Fig. 6B and C). Knockdown of *AHR* (Fig. 6D) reduced CNN1 expression by ~30% in skatole-treated cells (Fig. 6E) and attenuated the skatole-induced proliferation and migration of HASMCs by about 40% (Fig. 6F–H). Chromatin immunoprecipitation analysis further demonstrated that skatole exposure significantly enhanced AHR binding to the *CNN1* promoter compared with vehicle treatment (Fig. 6I). To directly evaluate the role of the AHR-binding site within the *CNN1* promoter, site-directed mutagenesis was performed based on the PROMO 3.0 prediction (Fig. S10B). Mutation of the putative AHR-binding region at −368/−378 bp (Mut) relative to the transcription start site reduced skatole-induced *CNN1* transcription by over 70% (Fig. 6J), confirming that AHR binding was essential for skatole-stimulated *CNN1* activation. Together, these results demonstrate that AHR is required for CNN1-dependent VSMC proliferation and migration induced by skatole.

## Discussion

**Figure 7:**
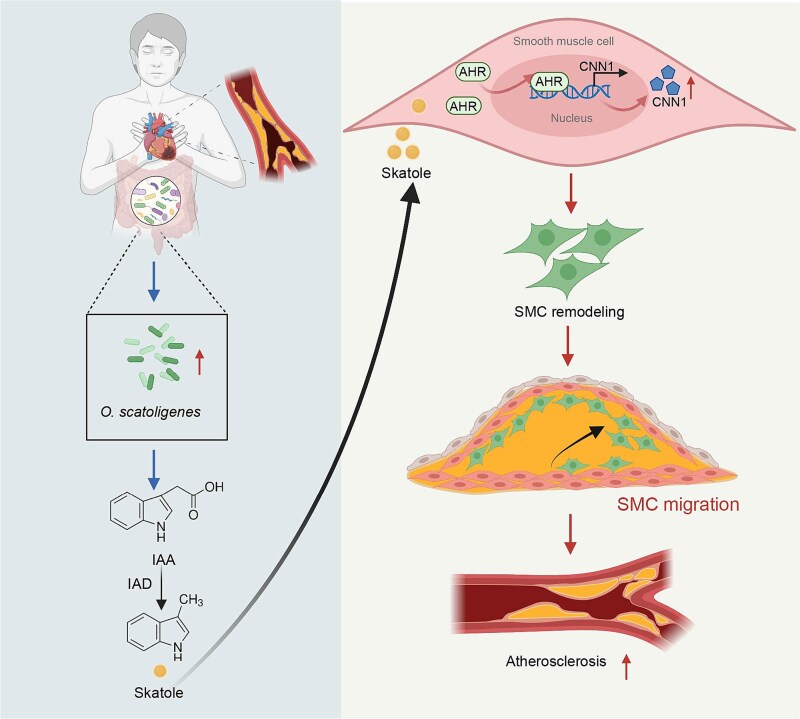
Schematic summary illustrating the potential mechanism whereby high *O. scatoligenes* promotes the progression of atherosclerosis. Integration of metagenomics and metabolomics is employed to interrogate the alterations of gut microbiota between obstructive CAD and non-CAD controls. The microbiome of patients with CAD is characterized by higher abundance and stronger interactions of *O. scatoligenes*. Increased skatole, a microbial product of tryptophan metabolism, serves as the main signaling transducer mediating the detrimental effect of *O. scatoligenes* on atherosclerosis, mainly through promoting the proliferation and migration of vascular smooth muscle cells in an AHR-CNN1-dependent manner. Created with biorender.

Through integrated metagenomic and metabolomic analyses in patients with CAD and non-CAD controls, we identified that elevated abundance of *O. scatoligenes* was independently associated with a greater atherosclerotic burden, and that skatole served as the key microbial effector linking *O. scatoligenes* to accelerated atherosclerosis. Administration of *O. scatoligenes* or skatole promoted atherosclerotic plaque formation by enhancing CNN1-mediated VSMC proliferation and migration. Mechanistically, increased skatole upregulated CNN1 transcription through activation of the AHR (Fig. 7). Collectively, these findings suggest a causal role of *O. scatoligenes* in exacerbating atherosclerosis.

Although altered gut microbiota has long been implicated in the pathogenesis of CAD, most previous studies were restricted to correlative analyses without mechanistic validation [21], leaving the underlying biology unresolved. Here, using both human cohorts and murine models, we provided evidence that *O. scatoligenes* functioned as a major bacterium driving CAD progression. By integrating differential abundance, co-occurrence network, and validation analyses, we identified *O. scatoligenes*, a strictly anaerobic, non-motile, and non-sporulating member of the family *Atopobiaceae* [22, 23], as closely associated with CAD and its severity. Consistent with our observation, the *Olsenella* genus was reported to be causally linked to metabolic syndrome in European populations [24], and enriched in murine models of non-alcoholic fatty liver disease [25], whereas the anti-obesity effect of probiotic-fermented tomato was accompanied by reductions in *Olsenella* abundance [26]. Recent studies further showed that *Olsenella* was positively associated with interferon α, a key cytokine contributing to vascular inflammation and atherosclerosis [27]. Here, we extended these findings by demonstrating *O. scatoligenes* gavage elevated SBP without affecting DBP, and aggravated atherosclerotic plaque formation in the absence of altered cholesterol and glucose metabolism [28]. The selective elevation in SBP aligned with prior evidence showing that SBP was more sensitive to early vascular alterations and served as a stronger predictor of CAD than DBP [29–31]. Mechanistically, *O. scatoligenes* promoted atherosclerosis primarily through enhancing VSMC proliferation and migration. However, we have to acknowledge the limitation of employing a type strain alone, further work using isolates from CAD patients is warranted to confirm the biological relevance of *O. scatoligenes* in atherosclerosis. Nevertheless, our findings highlight the potential of targeting *O. scatoligenes* for CAD prevention.

By combining metagenomic and metabolomic analyses, we characterized the metabolic alterations in patients with CAD. In line with previous reports describing disturbances in glycerophospholipid metabolism, starch and sucrose metabolism, bacterial secretion system, and amino acid metabolism in atherosclerosis [32–35], we identified skatole, an established microbial tryptophan fermentation product [36], as the molecular transducer mediating the atherogenic effects of *O. scatoligenes*. Microbial tryptophan metabolism exerted both beneficial and detrimental effects on cardiometabolic health depending on the abundance of key microbial enzymes. For example, in healthy individuals, enrichment of *Bacteroides fragilis* and *Bacteroides thetaiotaomicron* increased indole-3-acetic acid production [37], whereas enrichment of *Klebsiella pneumoniae* diverted tryptophan metabolism toward indoxyl sulfate synthesis, leading to ruptured intracranial aneurysms [38]. Extending these observations, we identified *O. scatoligenes*-derived skatole as an independent risk factor for aggravated atherosclerosis. In support of our finding, skatole had also been reported to promote inflammatory bowel disease by disrupting intestinal immune homeostasis [39], and tryptophan degradation was found to be elevated in patients with acute myocardial infarction and diabetes [40, 41]. Capable of encoding the rate-limiting enzyme of *iad* [42], currently *O. scatoligenes* remains the only known mammalian bacterium able to produce skatole [22, 23, 42, 43]. Consistently, mice colonized with *O. scatoligenes* exhibited increased fecal *iad* activity and elevated circulating skatole levels. The median plasma skatole concentration reached ~2 ng/ml in patients with severe CAD, comparable to levels observed in hepatic encephalopathy (5.3 ng/ml) [44], where skatole acted as a pulmonary toxin associated with acute pulmonary edema and emphysema [45, 46]. Moreover, consistent with our findings, incubation with skatole in hepatocytes was reported to induce cytotoxicity via activation of cytochrome P450 family 1 subfamily A member 1 [46]. Although dietary tryptophan intake may confound skatole levels in humans, our murine experiments, performed under identical diets, demonstrated that *O. scatoligenes* gavage increased plasma skatole by over 200% (Fig. 3H), underscoring the diet-independent contribution. Collectively, these findings support the concept that skatole represents an unfavorable microbial product of tryptophan degradation relevant to CAD.

Mounting evidence indicates that VSMC remodeling, characterized by increased proliferation and migration potential [4, 7, 47], is critically involved in all stages of atherosclerosis [7]. In this study, both *O. scatoligenes* and its metabolite skatole accelerated plaque formation mainly through enhancing VSMC proliferation and migration to the intima, supporting the notion that excessive VSMC proliferation drives atherosclerosis progression [8]. Similarly, 5-hydroxytryptamine, a dietary tryptophan derivative from chocolate liquor, was shown to exacerbate atherosclerosis through 5-hydroxytryptamine 2A receptor–dependent VSMC proliferation and migration [48, 49]. Conversely, polyphenols from mulberry and *Hibiscus* leaf protected against atherosclerosis by inhibiting abnormal VSMC proliferation and migration [50, 51], and moderate exercise similarly suppressed VSMC proliferation and mitigated vascular remodeling [52]. Furthermore, pharmacological agents such as PF-477736 [53], a selective Chk1 inhibitor, and V-9302 [54], had demonstrated anti-atherogenic effects in preclinical models primarily via inhibition of VSMC proliferation. In this context, our findings indicate that aberrant microbial metabolism provides an additional stimulus for VSMC dedifferentiation, and that suppressing VSMC proliferation and migration may represent a shared mechanism underlying dietary, pharmacological, and exercise interventions. However, VSMC remodeling is complex, and its precise contribution to atherosclerotic cardiovascular diseases remains incompletely understood. Consistent with reports showing increased CNN1 expression in advanced murine atherosclerotic lesions [7], our data revealed that *CNN1* downregulation in VSMCs largely abolished the pro-atherosclerotic effect of *O. scatoligenes* and skatole. Supporting this observation, CNN1 was found to inhibit VSMC contraction *in vitro* [55], and *Cnn1* knockout mice exhibited reduced vascular stiffness and contractile responses to phenylephrine [56]. Collectively, our results reinforce the crucial role of CNN1 in regulating VSMC remodeling. However, because only partial knockdown of *CNN1* was achieved, we cannot exclude the possibility that skatole may also act through other downstream AHR targets, such as fibroblast growth factor and cytochrome P450 1A1, to promote vascular remodeling [57, 58]. Moreover, given the diverse physiological roles of CNN1 in vascular biology [59], direct therapeutic targeting of CNN1 may not be optimal or safe. From a translational perspective, dietary or pharmacological inhibition of *O. scatoligenes* or its skatole-producing enzyme *iad* represents a more feasible and attractive strategy for CAD management.

In conclusion, our study identifies increased microbial skatole production as a central effector linking *O. scatoligenes* to enhanced atherosclerosis through activation of the AHR-CNN1 axis, which promotes VSMC proliferation and migration.

## Supplementary Material

SUPPLEMENTAL_MATERIAL-ISME-clean_version_wraf238

## Data Availability

The metagenomic sequencing data generated in this study have been deposited in the NCBI Sequence Read Archive (SRA Accession No. PRJNA1214590).
