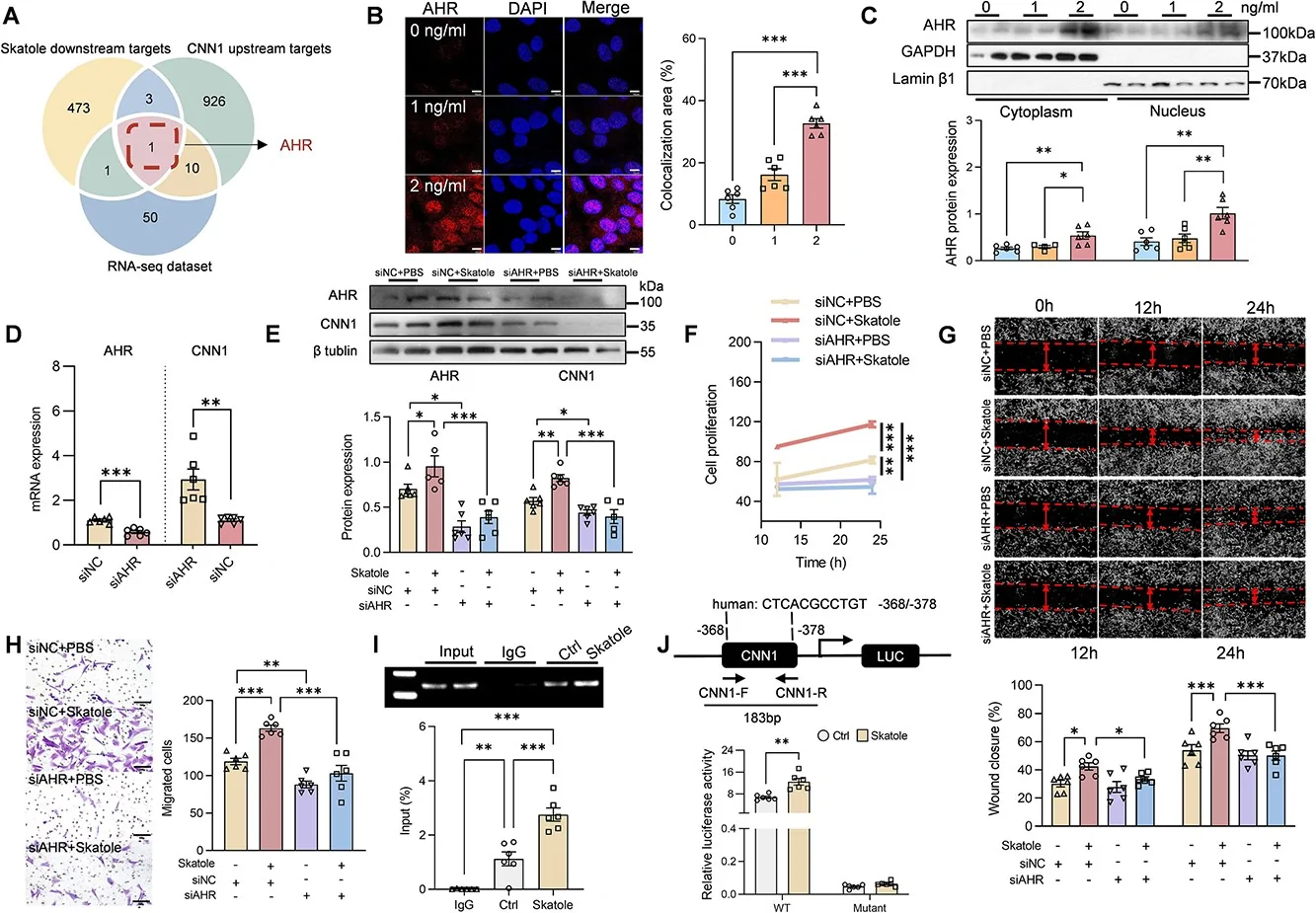# Correction to: *Olsenella scatoligenes*-derived skatole promotes smooth muscle cell proliferation and migration to aggravate atherosclerosis

**DOI:** 10.1093/ismejo/wrag240

**Published:** 2026-10-14

This is a correction to: Yawen Zhao, Jiarui Chen, Shanshan Zhu, Yingxi Xu, Jiangyuan Zhu, Jialu Yang, Weibin Zhou, Ying Yang, Maohuan Lin, Qian Chen, Min Xia, Yangxin Chen, Yan Liu, *Olsenella scatoligenes*-derived skatole promotes smooth muscle cell proliferation and migration to aggravate atherosclerosis, *The ISME Journal*, Volume 19, Issue 1, January 2025, wraf238, https://doi.org/10.1093/ismejo/wraf238

In the originally published version of this manuscript, the experimental results for the siAHR+PBS group were mistakenly presented as those for the siAHR+skatole group in Figure 6G.

This error has been corrected so that Figure 6G reads:


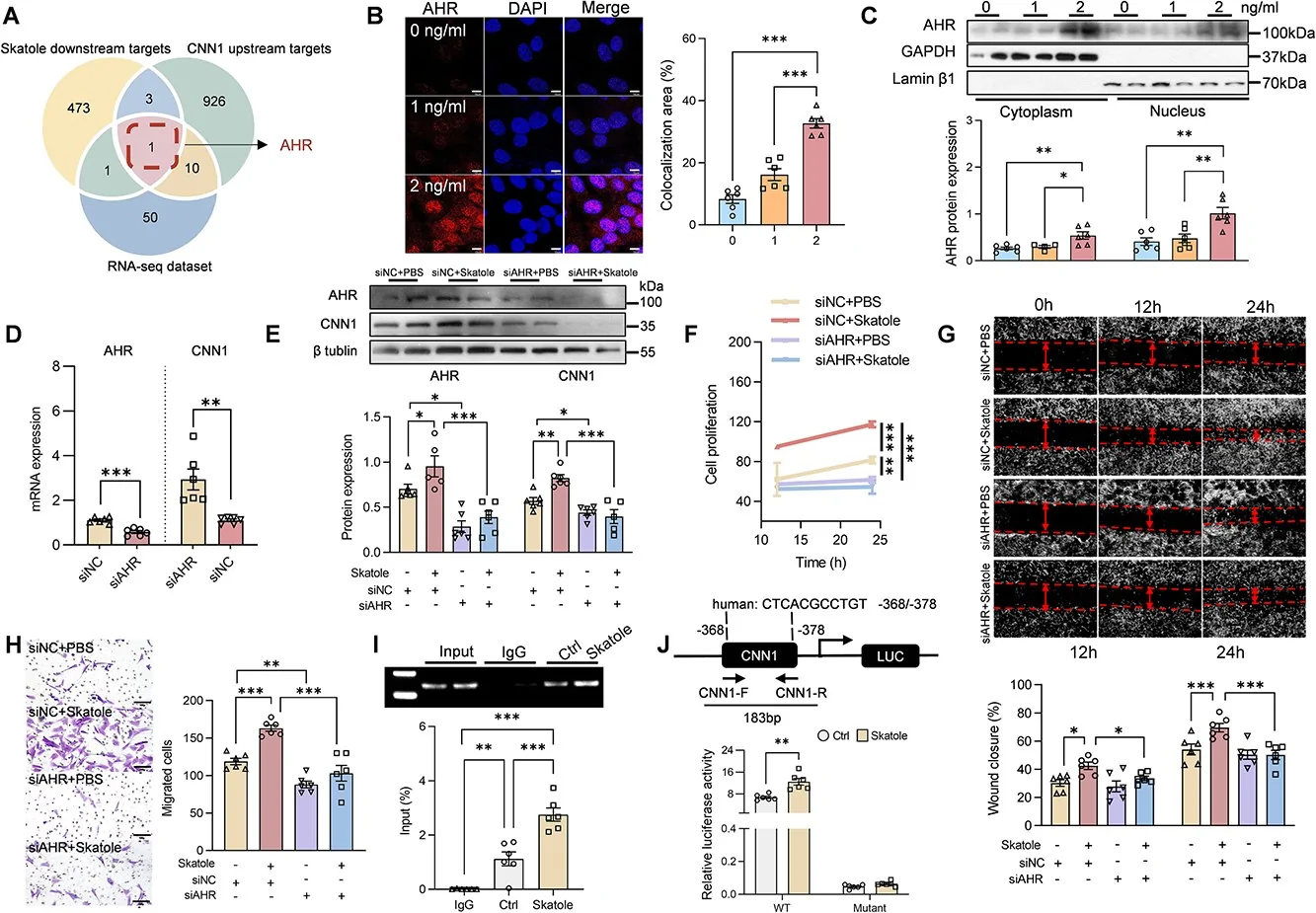


Instead of: