# Supplementary material for: Olsenella scatoligenes-derived skatole promotes smooth muscle cell proliferation and migration to aggravate atherosclerosis
Source: ISME J. 2025 Oct 23;19(1):wraf238. doi: 10.1093/ismejo/wraf238 (PMC12613830; doi:10.1093/ismejo/wraf238)
Supplement: SUPPLEMENTAL_MATERIAL-ISME-clean_version_wraf238 [file supplemental_material-isme-clean_version_wraf238.docx]

**SUPPLEMENTAL MATERIAL TO**

***Olsenella scatoligenes*-derived Skatole Promotes Smooth Muscle Cell Proliferation and Migration to Aggravate Atherosclerosis**

Yawen Zhao, PhD^1,#^, Jiarui Chen, PhD^1,#^, Shanshan Zhu, PhD^1^, Yingxi Xu, PhD^2^, Jiangyuan Zhu, PhD^1^, Jialu Yang, PhD^1^,Weibin Zhou, PhD^3,4^, Ying Yang, PhD^3,4^, Maohuan Lin, PhD^3,4^, Qian Chen, PhD^3,4^, Min Xia, PhD^1,*^, Yangxin Chen, PhD^3,4,*^, Yan Liu, PhD^1,5,*^

**Short title**: *Olsenella scatoligenes* and atherosclerosis

^1^Guangdong Provincial Key Laboratory of Food, Nutrition and Health, and Department of Nutrition, School of Public Health, Sun Yat-sen University, Guangzhou, P.R. China

^2^Guangdong Provincial Key Laboratory of Food, Nutrition and Health, and Department of Statistics and Epidemiology, School of Public Health, Sun Yat-sen University, Guangzhou, P.R. China

^3^Department of Cardiology, Sun Yat-sen Memorial Hospital, Sun Yat-sen University, Guangzhou, P.R. China

^4^Guangdong Province Key Laboratory of Arrhythmia and Electrophysiology, Sun Yat-sen Memorial Hospital, Sun Yat-sen University, Guangzhou, P.R. China

^5^Lead Contact

^#^These two authors contributed equally

^*^ Address for Correspondence: Prof. Yan Liu, Department of Nutrition, School of Public Health, Sun Yat-sen University, Public Health Building, 74, Zhongshan Road 2, Yuexiu District, Guangzhou, Guangdong, 510080, China. E-mail: [liuyan215@mail.sysu.edu.cn](mailto:liuyan215@mail.sysu.edu.cn); OR

Prof. Yangxin Chen, Department of Cardiology, Sun Yat-sen Memorial Hospital, Sun Yat-sen University, 107, Yanjiang West Road, Yuexiu District, Guangzhou, Guangdong, 510100, China. E-mail: [chenyx39@mail.sysu.edu.cn](mailto:chenyx39@mail.sysu.edu.cn); OR

Prof. Min Xia, Department of Nutrition, School of Public Health, Sun Yat-sen University, Public Health Building, 74, Zhongshan Road 2, Yuexiu District, Guangzhou, Guangdong, 510080, China. E-mail: [xiamin@mail.sysu.edu.cn](mailto:xiamin@mail.sysu.edu.cn).

**This file includes**

**1. Supplementary Materials & Methods**

**2. Figures S1-S10**

**3.** **Table S1-S5**

**Supplementary Materials & Methods**

**Study participants**

Participants in both discovery and validation cohorts were recruited from the Department of Cardiology, Sun Yat-sen Memorial Hospital in 2019 and 2021, respectively. Consecutive patients undergoing coronary angiography for suspected CAD were invited to join the study through posters and flyers.

*Inclusion* *criteria* were as follows: (1) local residents aged ≥18 years; (2) no severe disability, malignancy or cancer; (3) absence of advanced heart failure with left ventricular ejection fraction <40%; (4) no infections within past month; (5) no use of antibiotics, prebiotics, or probiotics during the 3 months prior to sample collection.

*Exclusion criteria* were as follows: (1) patients with acute coronary syndrome or those who had undergone percutaneous coronary intervention or coronary artery bypass graft surgery within past 6 months; (2) patients with biliary obstruction, acute or chronic cholecystitis, viral hepatitis (acute or chronic), liver cirrhosis, diarrhea, thyroid dysfunction (hyper- or hypothyroidism), chronic renal insufficiency, pregnancy, or gastrointestinal diseases; (3) psychiatric illness that impairing the ability to comprehend the nature, scope, and possible consequences of the study.

Coronary angiograms were independently reviewed by three experienced cardiologists. Obstructive CAD was defined as the presence of at least one stenosis ≥ 50%, whereas individuals with stenosis < 50% were categorized as non-obstructive CAD controls[1]. The Gensini score was calculated as previously described[2], and the SYNTAX score was determined according to the 2011 ACCF/AHA/SCAI guideline[3].

**Collection of biological samples and covariates**

Anthropometric measurements (body weight and height) and blood pressure were obtained by trained staff. Blood pressure was measured on the right arm with participants seated after a 10–15 min rest using a validated automatic digital sphygmomanometer (Omron HEM-7136). Trained interviewers conducted face-to-face interviews using a standardized questionnaire to collect demographic information, medical history, current medications, and lifestyle factors.

Type 2 diabetes mellitus was defined as fasting plasma glucose (FPG) ≥ 7.0 mmol/L, according to the 2019 American Diabetes Association guidelines[4].

Hypertension was defined as systolic blood pressure ≥ 140 mmHg or diastolic blood pressure ≥ 90 mmHg, according to the 2017 ACC/AHA Hypertension Guidelines[5].

Dyslipidemia was defined as triglycerides ≥ 200 mg/dL, total cholesterol ≥ 240 mg/dL, LDL-c ≥ 160 mg/dL, HDL-c ≥ 60 mg/dL or < 40 mg/dL for men and < 50 mg/dL for women, according to the 2018 ACC/AHA Guidelines on the Management of Blood Cholesterol[6].

Plasma samples were collected after overnight fasting and before coronary angiography. Routine biochemical parameters, including total cholesterol, triglycerides, HDL-c, and LDL-c, were measured using an automatic analyzer (Beckman Coulter chemistry analyzer AU5800; Beckman Coulter Co., Ltd, Tokyo, Japan). ApoB, NT-proBNP, hs-TnT, CK, and CK-MB were quantified on a fully automated electrochemiluminescence immunoassay system (Roche Cobas e601; Hoffmann‐La Roche Ltd, Basel, Switzerland). Fecal samples were collected using MGIEasy kits containing a stabilizing reagent to allow preservation at room temperature. All fecal samples were subsequently stored at -80℃ until further processing.

**Fecal sample collection and shotgun metagenomics sequencing**

Total DNA was extracted from frozen stool samples using MagMAX Microbiome Ultra Nucleic Acid Isolation Kit (Thermo Fisher Scientific, MA, USA) according to the manufacturer’s protocol. DNA concentration and purity were assessed using a NanoDrop 2000 spectrophotometer, and integrity was verified by electrophoresis on a 1% agarose gel. Approximately 350-bp DNA fragments were generated and subjected to paired-end library construction using the NEBNext Ultra DNA Library Prep Kit for Illumina (NEB, USA). After adaptor ligation, qualified libraries were pooled and sequenced on the NovaSeq 6000 Platform (Illumina, San Diego, CA, USA) using a paired-end 150 bp strategy. Each sample yielded approximately 8 Gb of sequencing data. Sequencing was conducted by Novogene Bioinformatics Technology Co., Ltd. (Beijing, China).

**Fecal metabolomics profiling in study participants**

Metabolomic profiling of fecal samples was performed using a widely targeted LC–MS/MS approach with minor modifications from previously described methods[7]. Briefly, 20 mg of fecal material was extracted in 400 μL of 70% methanol containing internal standards. After vortexing for 3 min, samples were sonicated in an ice bath for 10 min, vortexed again, and then incubated at –20°C for 30 min to promote precipitation. Following centrifugation at 12,000 rpm for 10 min at 4°C, the supernatant was collected and centrifuged again under the same conditions for 3 min. A 200 μL aliquot of the clarified extract was used for LC–MS/MS analysis. Chromatographic separation was carried out on an ACQUITY UPLC HSS T3 C18 column (1.8 μm, 2.1 × 100 mm; Waters) using a Shim-pack UFLC system (Shimadzu). Mass spectrometric detection was performed on a QTRAP 6500 + system (Sciex) equipped with an electrospray ionization source operating in both positive and negative ion modes. Source parameters were set as follows: GSI 55 psi, GSII 60 psi, CUR 25 psi, CAD high, source temperature 500°C, and ion spray voltage + 5500 V (positive mode) or − 4500 V (negative mode). Calibration and tuning were performed using polypropylene glycol solutions. Multiple reaction monitoring (MRM) transitions were applied for metabolite quantification, with data acquisition and processing conducted using Analyst 1.6.3 software (Sciex).

**Targeted detection of circulating skatole in both humans and mice**

Skatole levels in both human plasma and mouse serum samples were quantified using gas chromatography-mass spectrometry (GC–MS). Plasma samples (100 μL) or standard solution (100 μL) were mixed with 300 μL of acetonitrile containing 0.01% formic acid (v/v = 9:1), and internal standard skatole-D_8_ was added at a concentration of 25 ng/μL. After vortexing for 15 s, proteins were precipitated by centrifugation at 15,000 rpm and 4℃ for 15 min. The supernatants were then transferred and filtered into vials for GC–MS analysis.

GC–MS analysis was performed on an Agilent 8890-5977B GC–MS system operating in ion scan mode (scan range: m/z 50–700). Samples were separated on a HP-5MS column (Agilent 19091S-433; 30 m × 0.25 mm ID × 0.25 μm df). The injector was operated in splitless mode, and the injector temperature was maintained at 250°C. Helium served as the carrier gas at a constant flow rate of 3 mL/min. The oven temperature program for the HP-5MS column was as follows: ramp from70°C to 250°C at 40°C/min, then held for 5 min. MS spectrometric detection was carried out in single ion monitoring (SIM) mode. The mass-to-charge ratios of the precursor-to-product ion reactions monitored (m/z) was: 130.0/77.0 for skatole. The analytical response was linear across the concentration range of 0.1–100 ng/mL. Raw MS data were processed using Mass Hunter software (Agilent, version B.08.00).

**Determination of lipid and blood pressure in mice**

Serum total cholesterol, triglycerides, HDL-c, and LDL-c were measured using commercial assay kits according to the manufacture’s instruction (Nanjing Jiancheng Bioengineering Institute, Nanjing, China**)**. Systolic and diastolic blood pressure were recorded in conscious mice using a tail-cuff system (BP-2010A; Softron Biotechnology)[8]. Mice were acclimated to the restrainers and placed on a warming platform to stabilize body (skin and tail) temperature at 37°C in a quiet environment. Measurements were taken over 2–3 consecutive days by the same investigator, and values were averaged from at least six replicates per session under resting conditions.

**Quantitative analysis of atherosclerotic lesions in mice**

After euthanasia, the entire aorta with the attached heart was carefully excised. The upper portion of the heart containing the aortic root was embedded in OCT compound (Sakura Finetek Japan, Tokyo, Japan) and cryosectioned at 8 μm thickness. Sections were stained with Oil Red O (Sigma-Aldrich, St. Louis, USA) to visualize lipid deposition and counterstained with hematoxylin (Servicebio, Beijing, China). After removal of adventitial adipose tissue, the remaining aorta was opened longitudinally, stained with Oil Red O, and lesion burden was quantified. Lesion area and size were calculated using ImageJ software (National Institutes of Health, Baltimore, MD, USA).

**Real-time quantitative PCR analysis**

Total RNA was extracted from aortic tissues and cultured cells using Trizol reagent (Invitrogen, CA, USA). Reverse transcription was performed with 500 ng of RNA using the 5 ×All-In-One qRT SuperMix (Vazyme, Nanjing, China). Quantitative real-time PCR was conducted on a QuantStudio 7 Real-Time PCR System (Applied Biosystems) using Universal SYBR qPCR Master Mix (Vazyme, Nanjing, China). The primer sequences used in this study are listed in **Table S3**.

**Immunohistochemical and immunofluorescence staining**

For immunohistochemistry, aortic root sections were fixed with 4% paraformaldehyde, treated with 3% hydrogen peroxide for 15 min to quench endogenous peroxidase activity, and incubated overnight with anti-SMA (1:100; Proteintech 67735, Wuhan, China). After incubation with a horseradish peroxidase–conjugated secondary antibody, staining was visualized using 3,3-diaminobenzidine, and nuclei were counterstained with Harris hematoxylin (Servicebio, China). Masson’s trichrome staining was performed according to the manufacturer’s instructions. Positive staining was quantified using Fiji ImageJ software and expressed as percentage of the total aortic area.

For immunofluorescence, aortic roots embedded in Tissue-tek OCT compound (Sakura Finetek Japan, Japan) were cut into 8 μm sections and blocked with goat serum at room temperature for 10 min. Slides were then incubated overnight at 4℃ with primary antibodies against Ki67 (1:100; Proteintech 27309, Wuhan, China), CNN1 (1:100; Proteintech 13938, Wuhan, China), AHR (1:100; Proteintech 28727, Wuhan, China), MOMA2 (1:400; Abcam ab33451, England) or IL-1β (1:200, Proteintech 26048, Wuhan, China) followed by incubation with fluorescently labeled secondary antibodies for l 1h at room temperature in the dark. Images were acquired using a Zessie LSM900 confocal microscope and processed with ImageJ Fiji software.

**Assessment of cell proliferation and migration**

To evaluate proliferation, HASMCs were seeded in 96-well plates and starved for 12 h in serum-free DMEM. Cells were then exposed to skatole for 12 or 24 h. Proliferative activity was quantified using the Cell Counting Kit-8 (CCK-8) assay (10 μL/well; APExBIO, USA), according to the manufacturer’s instructions.

For migration assays, HASMCs were seeded in 12-well plates and serum-deprived for 12 h, followed by treatment with graded concentrations of skatole for additional 24 h. Cell migration was assessed by both wound-healing and Transwell assays. For the wound-healing assay, a sterile pipette tip was used to create a perpendicular scratch in the cell monolayer. For the Transwell assay, cells were seeded in the upper chamber inserts (8 μm pore size; Corning, NewYork, USA). After incubation with skatole, adherent cells on the membrane were fixed with 4% paraformaldehyde and stained with crystal violet (Beyotime Biotechnology, Shanghai, China). Images were acquired using a light microscope (Zessie, Axiolab 5).

**Western blotting**

Total protein was extracted using RIPA buffer (Beyotime Biotechnology, Shanghai, China) supplemented with 1% protease and phosphatase inhibitors (Beyotime Biotechnology, Shanghai, China). Nuclear and cytoplasmic proteins were isolated using a commercial extraction kit (Beyotime Biotechnology, Shanghai, China). Proteins were separated by 10% SDS-PAGE and transferred onto PVDF membranes (Millipore Corp, Billerica, MA, USA). Membranes were blocked with 5% nonfat milk in PBST at room temperature for 2 h, followed by overnight incubation at 4℃ with primary antibodies against CNN1 (1:2000; Proteintech13938, Wuhan, China), AHR (1:2000;Proteintech28727, Wuhan, China), GAPDH (1:50000; Proteintech60004-1, Wuhan, China), Lamin B1 (1:20000; Proteintech66095, Wuhan, China) or β-tublin (1:10000; Affinity T0023, Jiangsu, China). After washing, blots were incubated with horseradish peroxidase–conjugated secondary antibodies for 2 h at room temperature. Protein bands were visualized using enhanced chemiluminescence (ECL, Thermo Fisher Scientific, Waltham, MA, USA).

**Small interfering RNA-mediated gene silencing**

To knock down target genes in HASMCs, small interfering RNAs (siRNAs) were designed and synthesized by Ribobio (Guangzhou RiboBio Co., Ltd.,China). HASMCs were transfected with siRNAs targeting *CNN1* or *AHR* using Lipofectamine 3000 (Thermo Fisher Scientific, Waltham, MA, USA) according to the manufacturer’s instructions. After 24 h of transfection, cells were treated with skatole at the indicated concentrations or with PBS as a control for another 24 h. A non-specific siRNA control (NC siRNA) served as the negative control.

**Chromatin immunoprecipitation**

Chromatin immunoprecipitation (ChIP) assays were performed in HASMCs treated with or without skatole for 24 h using a ChIP assay kit (Thermo Fisher Scientific 26157, Waltham, MA, USA) according to the manufacturer’s instructions. Briefly, cells were cross-linked with 1% formaldehyde for 20 min, quenched with glycine, lysed, and sonicated to shear chromatin Samples were then incubated overnight at 4℃ with an anti-AHR antibody (1:100; Proteintech28727, Wuhan, China) or normal IgG antibody as a negative control. Immunocomplexes were captured with magnetic beads and reverse cross-linked at 65℃ for 1.5 h in high-salt solution. Purified DNA was analyzed by real-time PCR using primers listed in **Table S3**. PCR products were separated on a 1.8% agarose gel and stained with GelRed, with 10% input DNA used for normalization.

**Luciferase reporter assay**

Potential AHR binding motifs within the promoter region of the *Cnn1* gene (-388/-378 bp) were predicted using the PROMO 3.0 (<http://alggen.lsi.upc.es/cgi-bin/promo_v3/promo/promoinit.cgi?dirDB=TF_8.3>). A mutant construct disrupting the predicted AHR-binding site (-378/-368 bp) was generated and cloned into the pGL3-basic vector via *kpnI* and *xhoI* restriction sites. The luciferase reporter plasmids and control plasmids were synthesized by Wuhan GeneCreate Biological Engineering Co., Ltd. HEK293T cells were co-transfected with the *Cnn1* luciferase reporter, AHR expression plasmid and pRL-TK vector using Lipofectamine 3000 transfection reagent (Thermo Fisher Scientific, Waltham, MA, USA). After 24 h of transfection, cells were treated with skatole or PBS control for an additional 24 h. Luciferase activities were quantified using the Dual-Luciferase Reporter Assay System (Promega, Shanghai, China).

**Statistical analysis of metagenomics and metabolomics**

***Quality control, taxonomic profiling, and functional annotation***

Raw shotgun metagenomic reads were processed for quality control through several steps. Human contaminant sequences were identified and removed by mapping reads to the UCSC *hg38* human reference genome using BWA-MEM. Subsequent preprocessing employed an *in-house* script to remove adapter sequences, low-quality reads, and PCR duplicates, as previously described[9]. High-quality clean reads were used for taxonomic profiling with MetaPhlAn 3.0 under default parameters[10]. Functional annotation of metagenomic data was performed using the HUMAnN3 pipeline[10]. KEGG Orthology groups (KOs) were annotated against the Kyoto Encyclopedia of Genes and Genomes (KEGG) database and normalized to copies per million (CPM). Unmapped and unintegrated read masses were excluded from downstream analyses.

***Microbial diversity analysis***

Alpha diversity, represented by the Shannon index, was calculated at the species level using the *vegan* package in R. Differences in alpha diversity between groups were evaluated with the Wilcoxon rank-sum test[11]. Beta diversity was assessed using Bray-Curtis dissimilarity, also computed with the *vegan* package in R, to characterize differences in community composition at the species level. Distance-based redundancy analysis (dbRDA) was performed with the *vegan* package to visualize microbial community structures. Permutational multivariate analysis of variance (PERMANOVA), implemented via the adonis function in *vegan*, was applied to test the statistical significance of group differences.

***Differential analysis for microbial features***

Differentially enriched microbial features were identified using the R package *MaAsLin2*. Analyses were performed on species-level abundance data using a compound Poisson linear model (CPLM), on microbial functional profiles using a linear model applied to log-transformed data, and on metabolomic data using a linear model. All models were adjusted for sex, age, BMI, and medication use. An FDR-adjusted *P* value < 0.2 was considered statistically significant[12].

***Co-abundant network and correlation analysis***

Co-abundance networks were constructed separately for obstructive CAD patients and non-CAD controls based on pairwise Spearman correlations among microbial species. Only statistically significant correlations (permutation-adjusted *P* < 0.2) with an absolute Spearman’s ρ > 0.2 were retained for visualization using the *ggraph* package in R. Network topological properties—including clustering coefficient, node degree, betweenness, and closeness—were computed with *igraph* and compared between groups by Student’s *t*-tests. Significant differences were defined at an FDR-adjusted *P* < 0.05. Networks were further partitioned into modules using the fast-greedy clustering algorithm in *igraph*, and hub species were defined as those with the highest node degree within each module.

To examine the association between the relative abundance of *O. scatoligenes* and cardiometabolic parameters, multivariate linear regression model was fitted with adjustment for sex, age, BMI, and medication use in both discovery and validation cohorts. Results with *P* < 0.05 were considered statistically significant. Associations between *O. scatoligenes* abundance and significantly altered pathways or metabolites identified in the discovery cohort were further evaluated using partial Spearman correlation analyses adjusted for sex, age, BMI, and medication use. Correlations with permutation-adjusted *P* < 0.2 were considered significant.

To compare plasma skatole levels between CAD and non-CAD participants, multivariate regression analyses were performed with adjustment for sex, age, BMI, and medication use in both the discovery and validation cohorts. Results with *P* < 0.05 were considered significant. The relationship between plasma skatole concentrations and CAD severity was assessed using partial Spearman correlation analyses with the Gensini score and SYNTAX score, adjusting for sex, age, BMI, and medication use in each cohort. Associations with *P* < 0.05 were regarded as statistically significant.

**RNA-sequencing and analysis**

Total RNA was isolated from mouse aortas using TRIzol reagent (Invitrogen, CA, USA) according to the manufacturer’s instructions. Following ribosomal RNA depletion and library preparation, sequencing was performed on the NovaSeq 6000 Platform (Illumina). Raw reads were processed with the same quality control pipeline used for metagenomic data. Clean reads were aligned to the mouse reference genome (*GRCm38*) and quantified as transcripts per million (TPM) using Salmon v0.8.2[13]. Expression matrices were imported into R using the *tximport* package[14].

Differentially expressed genes (DEGs) were identified using DESeq2, with significance thresholds set at FDR-adjusted *P* < 0.2 and |log2 fold change| > 1. Gene Ontology (GO) enrichment analysis of significant DEGs was performed using *clusterProfiler*. Genes involved in smooth muscle cell proliferation pathways (GO:0048660, GO:0048659, GO:0045446) were extracted to construct a correlation network. Significant gene–gene correlations (FDR-adjusted *P* < 0.1) were incorporated into the network, and topological parameters (degree, betweenness, and closeness) were calculated using *igraph*. For visualization, only the top 10 genes ranked highest in each property were displayed.

**
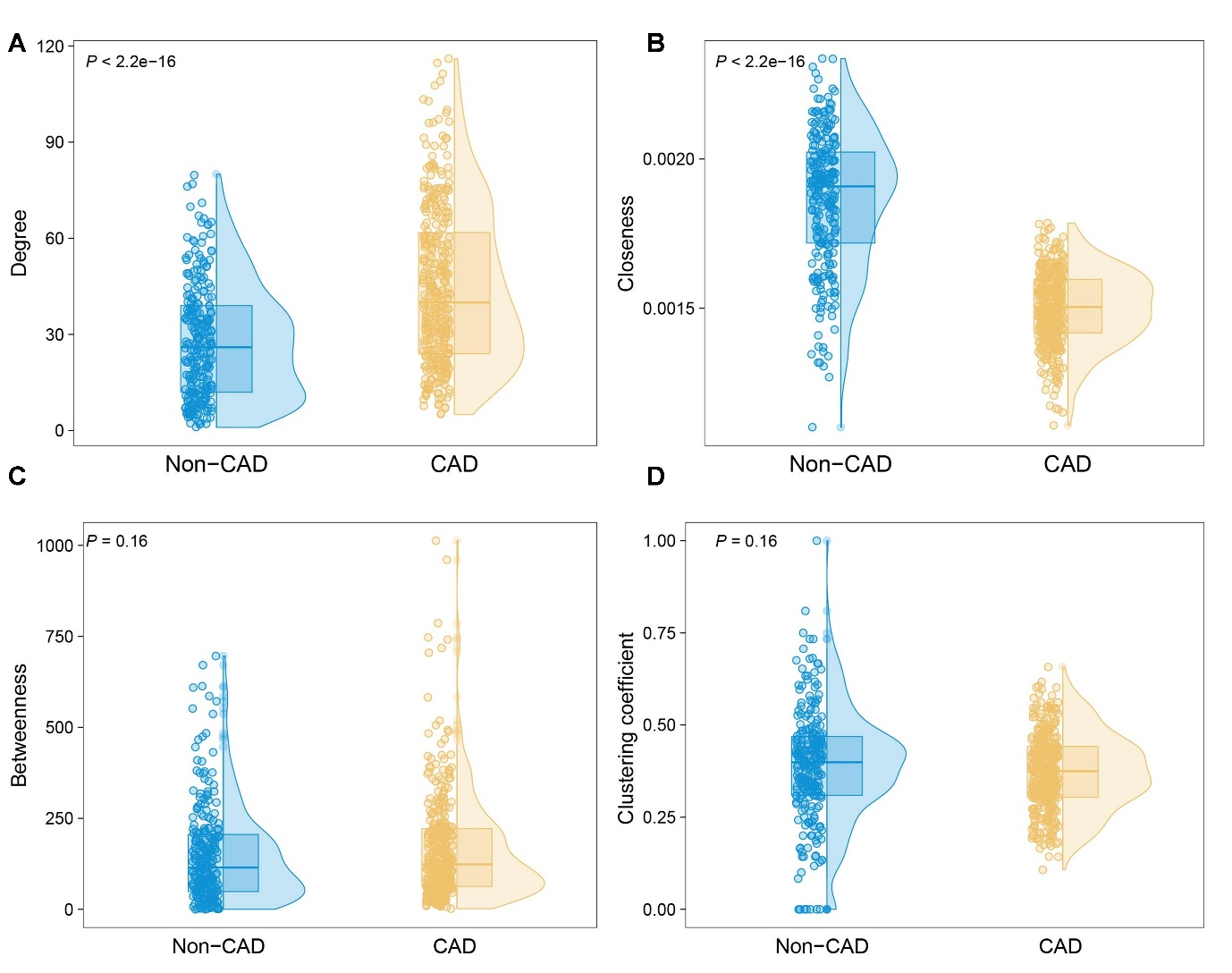
**

**Figure S1 Properties of the co-abundance network in patients with CAD and non-CAD controls.** (A) Node degree, (B) node closeness, (C) node betweenness and (D) clustering coefficient. All four network properties were compared using Student’s *t*-test. Results with adjusted *P* < 0.05 were considered statistically significant.

**
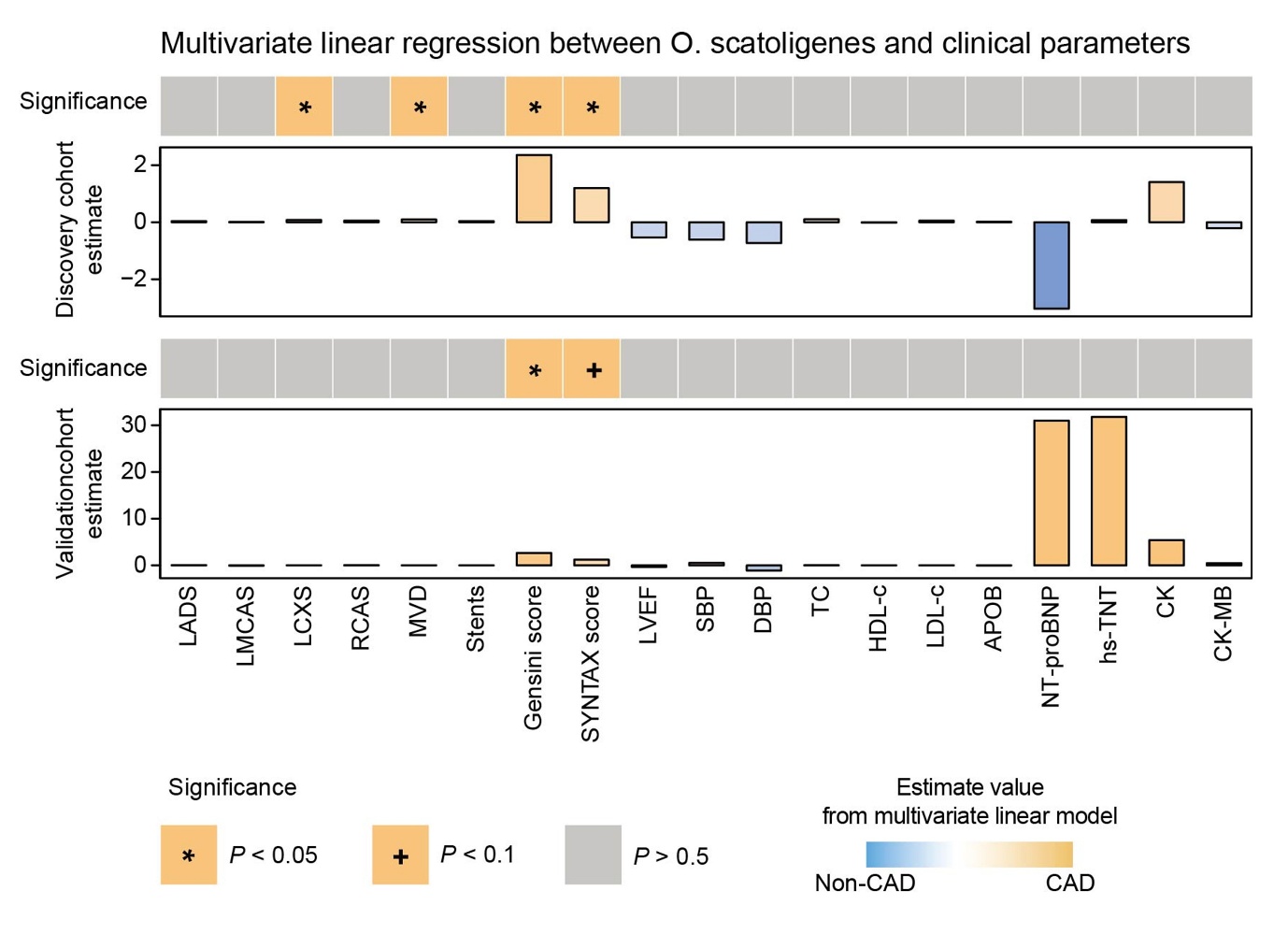
**

**Figure S2 Association of *O. scatoligenes* with cardiometabolic traits across cohorts.** A heatmap and bar plot summarizing the estimated coefficients between *O. scatoligenes* abundance and various clinical indices in the discovery (*upper panel*) and validation (*lower panel*) cohorts. Results were obtained from linear regression adjusted for age, sex, BMI, and medication use. The color intensity in both heatmap and the bar plot represents the strength and direction associations, with orange indicating positive and blue indicating negative associations. Asterisks denoted *P* < 0.05, and plus signs denoted *P* < 0.1.

**
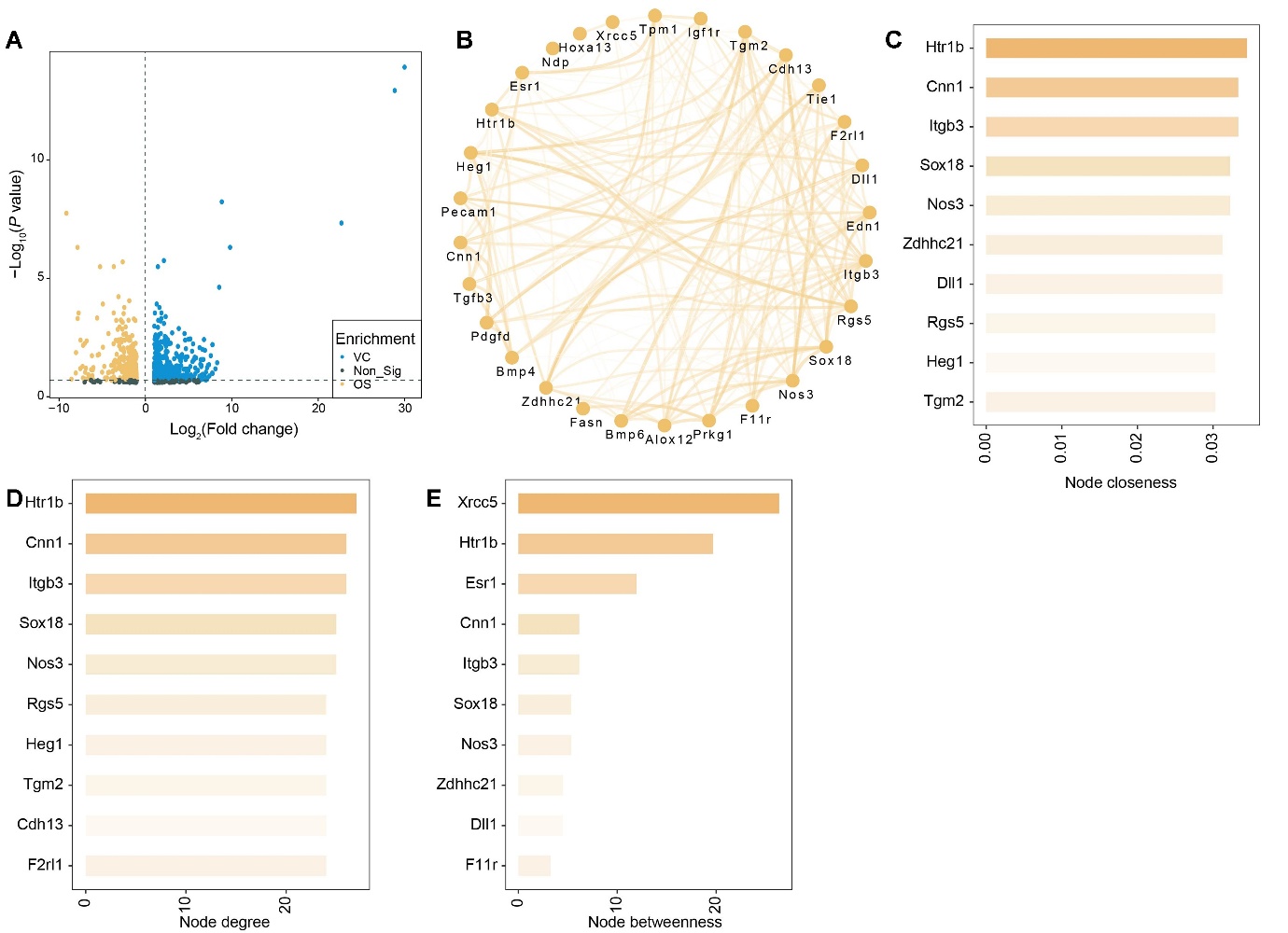
ZFigure S3 *CNN1* is the key target of *O. scatoligenes*.** (A)Volcano plot of differentially expressed genes in the aorta of mice gavaged with *O. scatoligenes* or vehicle control. (B) Network of differentially expressed genes. (C) Node degree, (D) betweenness and (E) closeness of each gene in the network. *P* values were calculated using a negative binomial general linear model. Differentially expressed genes with FDR-adjusted *P* < 0.2 and |log_2_(Fold change)| > 1 were considered as significant.

**
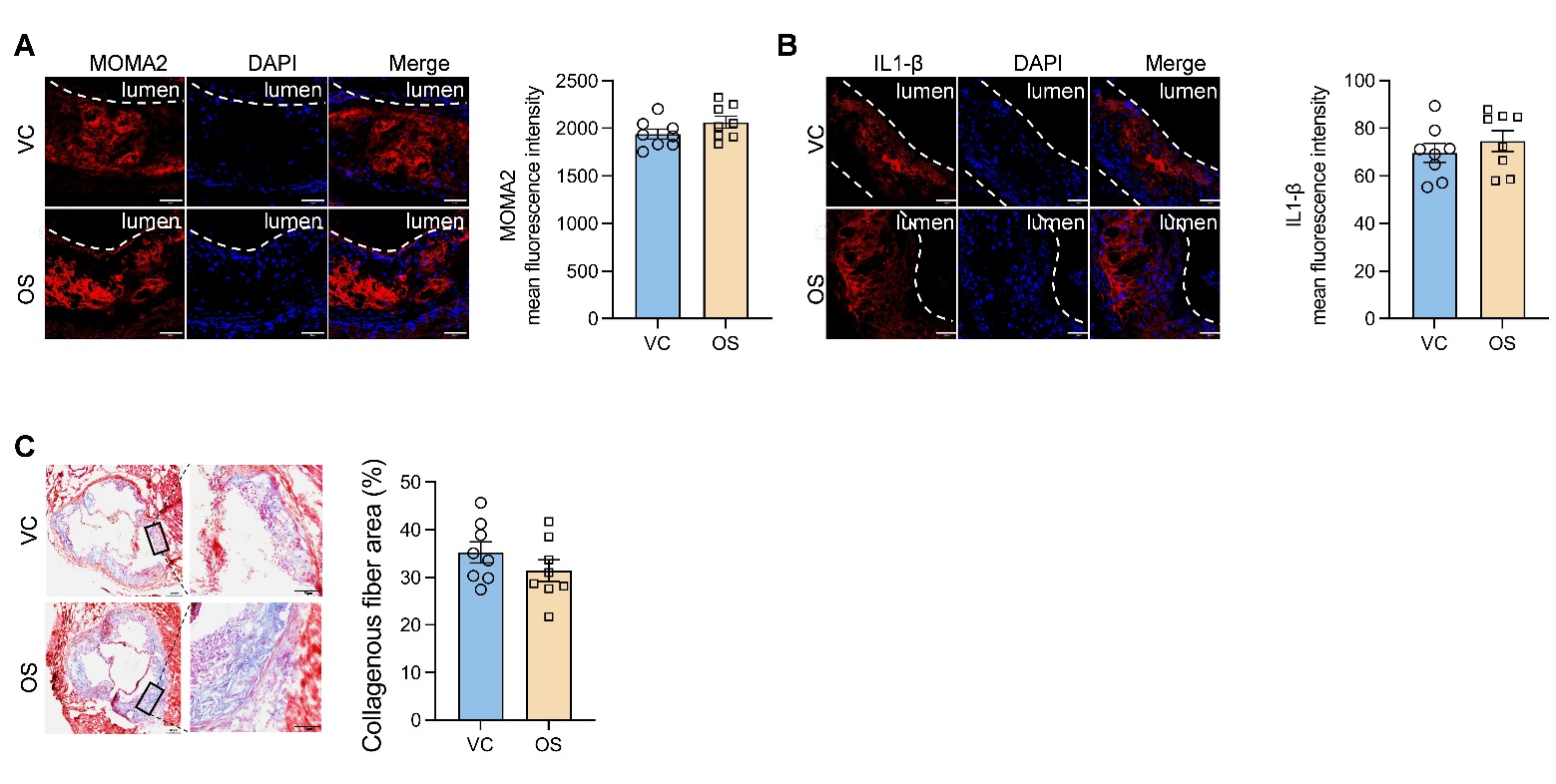
Figure S4. Macrophage infiltration, inflammation and collagen deposition in mice gavaged with *O. scatoligenes*.** Immunofluorescent staining of (A) MOMA2, and (B) IL1-β in the aortic root (scale bar, 50 μm). (C) Masson’s trichrome staining of the aortic root (scale bar, 200 μm and 50 μm, respectively). *P* values were determined by Student’s *t* test. Data were shown as mean ± SEM (n = 8 mice per group).

**
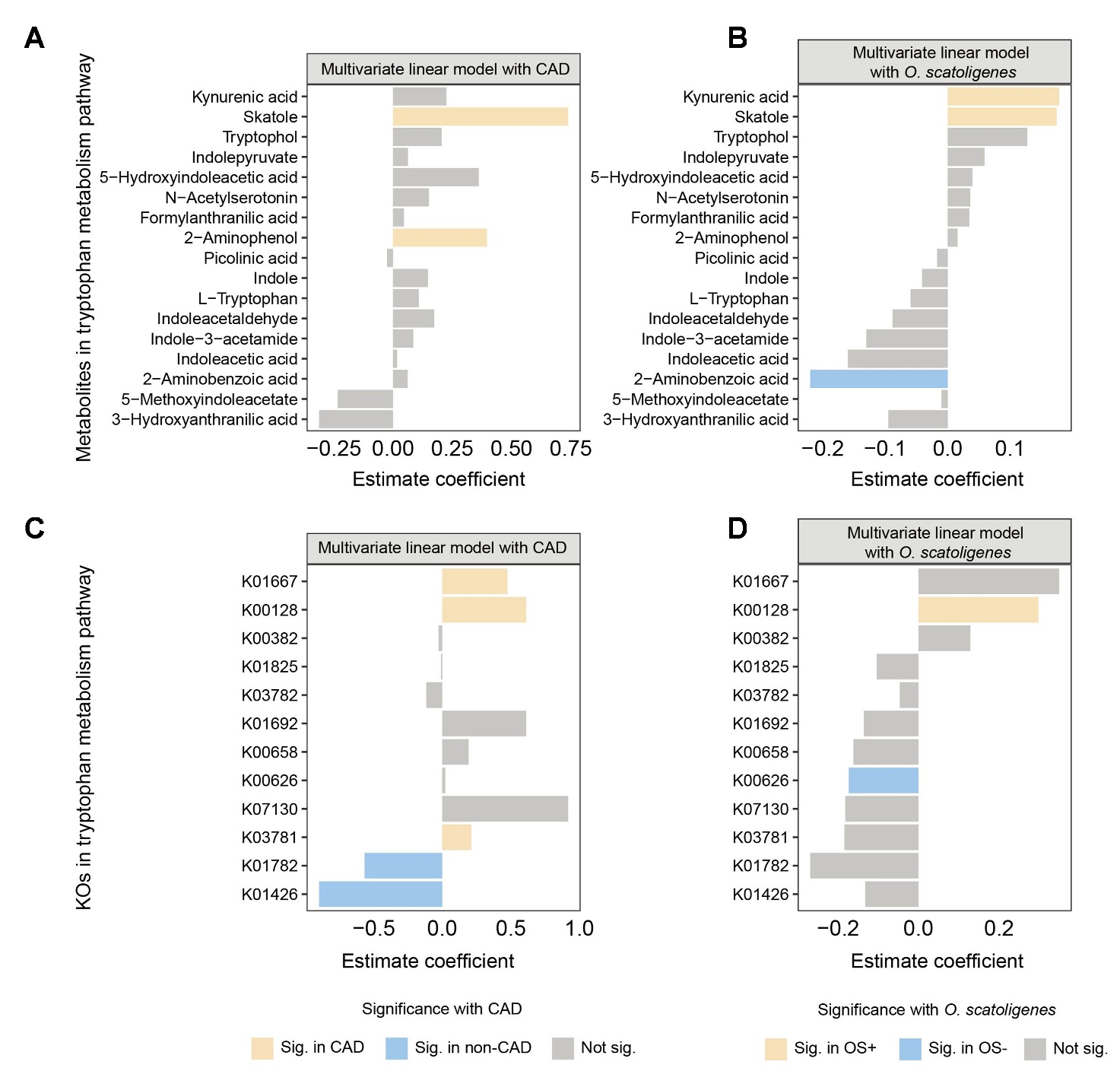
Figure S5. Association of metabolites and enzymes involved in tryptophan metabolism with CAD and *O. scatoligenes*.** Bar plots showing associations of tryptophan metabolism–related metabolites with (A) CAD status and (B) *O. scatoligenes* abundance and associations of microbial enzymes with (C) CAD status and (D) *O. scatoligenes* abundance. Estimated coefficients were derived from MaAsLin2 analysis adjusted for age, sex, BMI, and medication use. Orange and blue bars indicated significant positive and negative associations, respectively (*P_adj_* < 0.05), and gray bars represented non-significant associations.


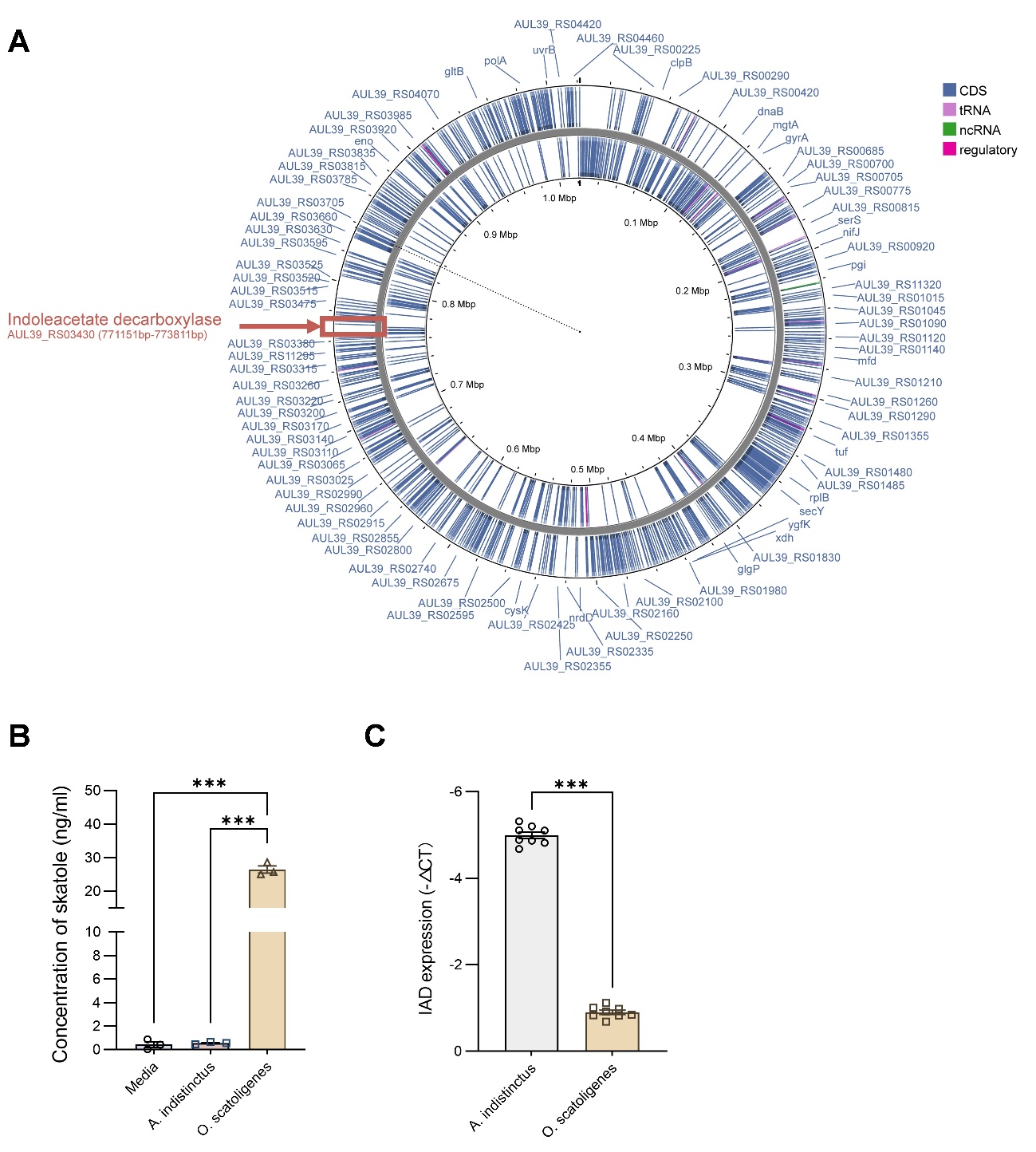


**Figure S6. Indoleacetate decarboxylase encoded in *O. scatoligenes*.** (A) Whole-genome sequence of *O. scatoligenes* and the gene encoding indoleacetate decarboxylase (*iad*). (B) Relative concentration of skatole in the supernatant of *O.scatoligenes* and *A. indistinctus* (negative control), normalized to culture medium. (C) Relative abundance of *iad* in *O.scatoligenes* and *A. indistinctus*. *P* values were determined by one-way ANOVA with Tukey’s test for B and by independent Student’s *t* test for C. Data were shown as mean ± SEM (n = 6 independent experiments). ^***^*P* < 0.001.

**
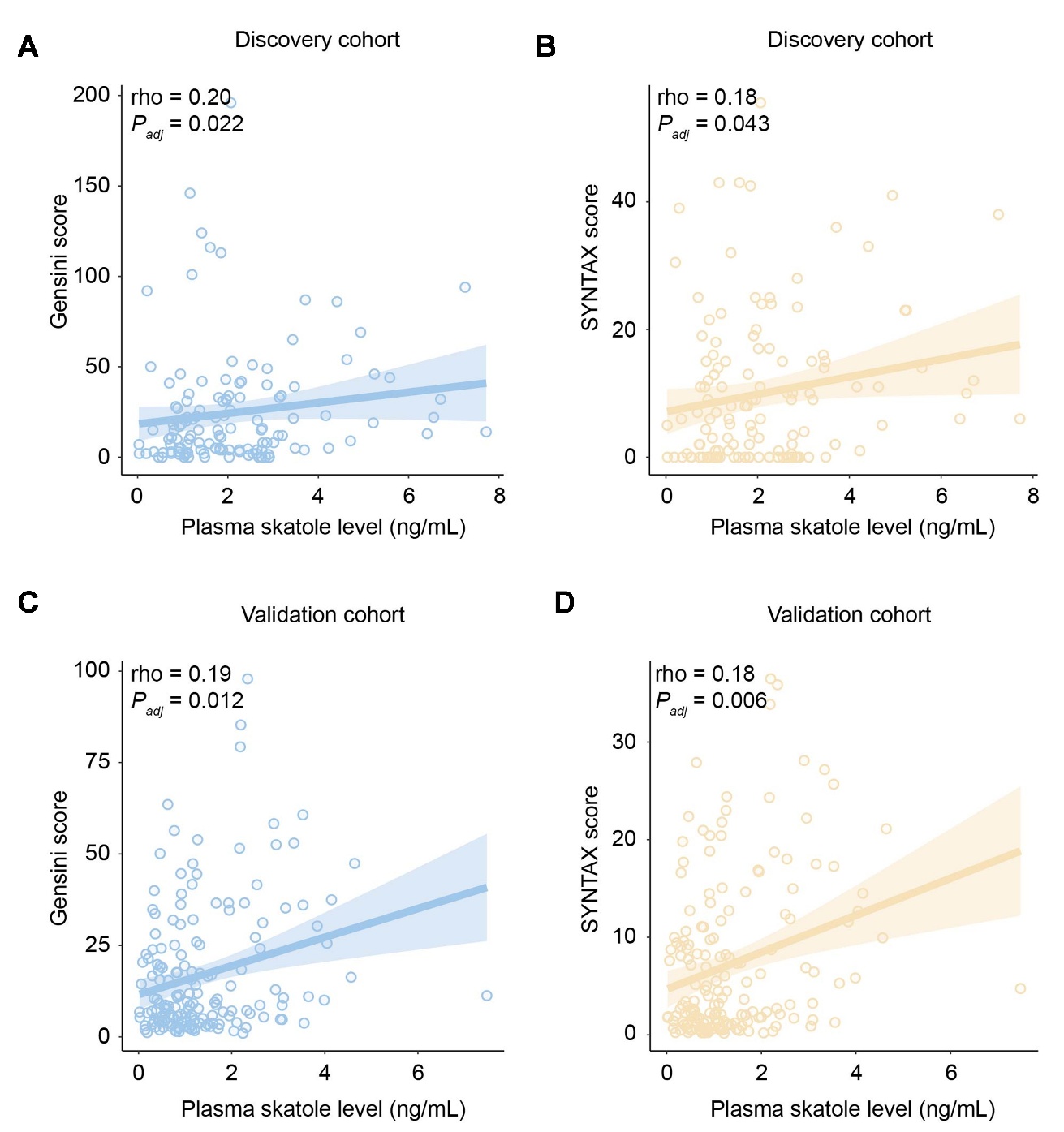
**

**Figure S7. Association of plasma skatole with CAD severity.** Scatter plots showing the association of plasma skatole with (A) Gensini score and (B) SYNTAX score in the discovery cohort, and with (C) Gensini score and (D) SYNTAX score in the validation cohort. *P* values were determined by partial Spearman correlation adjusted for age, sex, BMI and medication use.

**
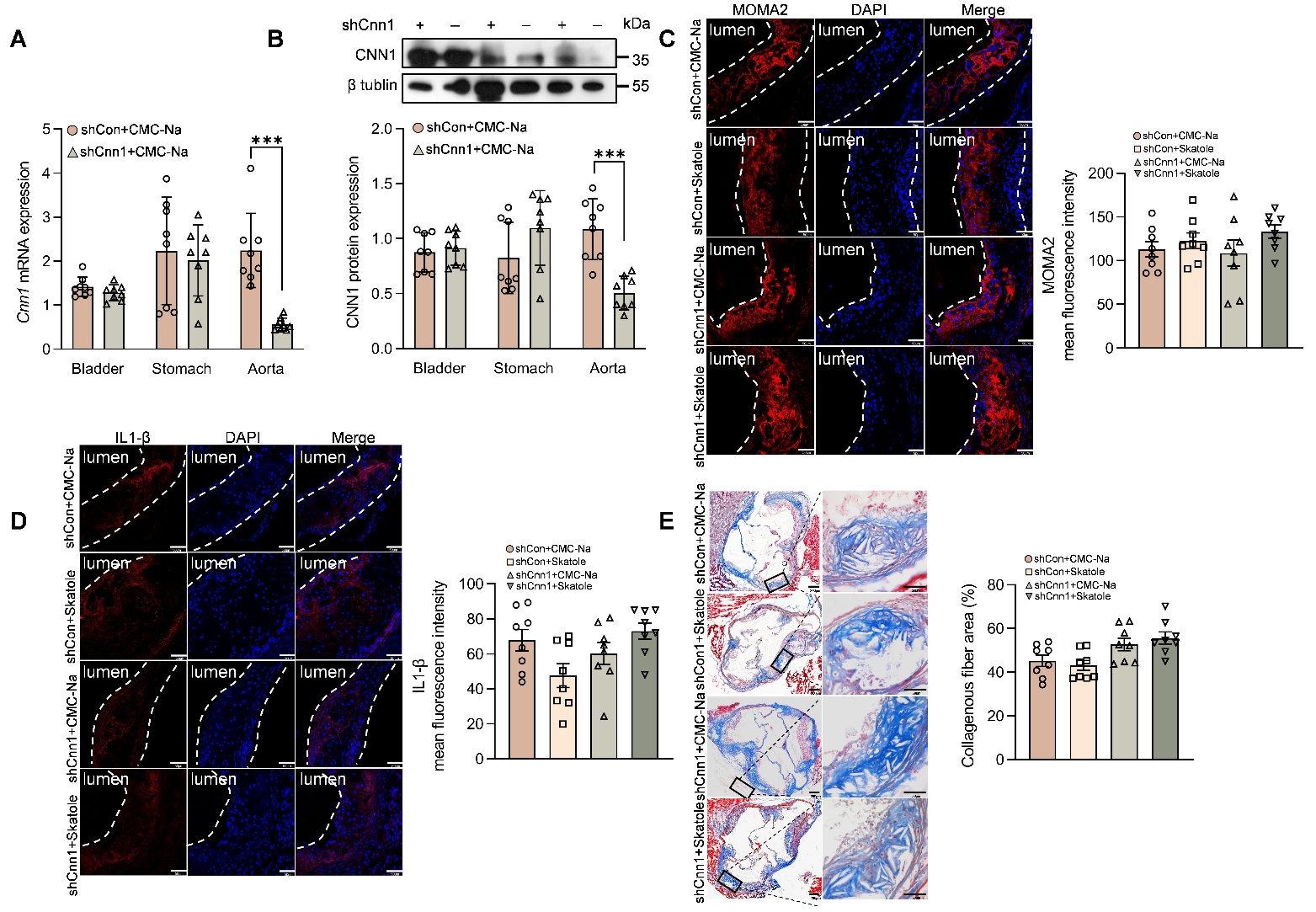
Figure S8. Macrophage infiltration, inflammation and collagen deposition in mice treated with skatole.** CNN1 expression at (A) mRNA and (B) protein level in bladder stomach and aorta. Immunofluorescent staining of (C) MOMA2 and (D) IL1-β in the aortic root (scale bar, 50 μm). (E) Masson’s trichrome staining of the aortic root (scale bar, 200 μm and 50 μm, respectively). *P* values were determined by Student’s *t* test for A-B, and two-way ANOVA for C-D. Data were shown as mean ± SEM (n = 8 mice per group). ^***^*P* < 0.001.


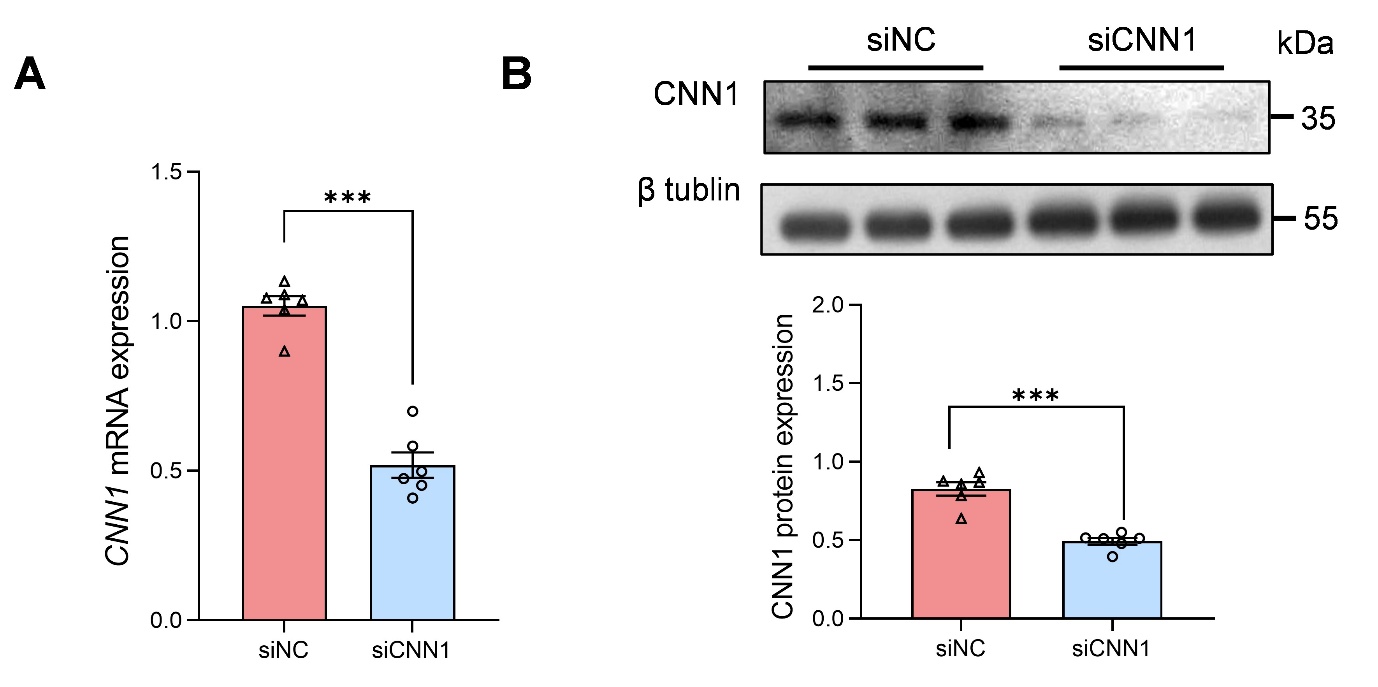


**Figure S9**. ***CNN1* silencing effect in HASMCs.** CNN1 expression at (A) mRNA and (B) protein levels. *P* values were determined by independent Student’s *t* test. Data were shown as mean ± SEM (n = 6 independent experiments). ^***^*P* < 0.001.

**
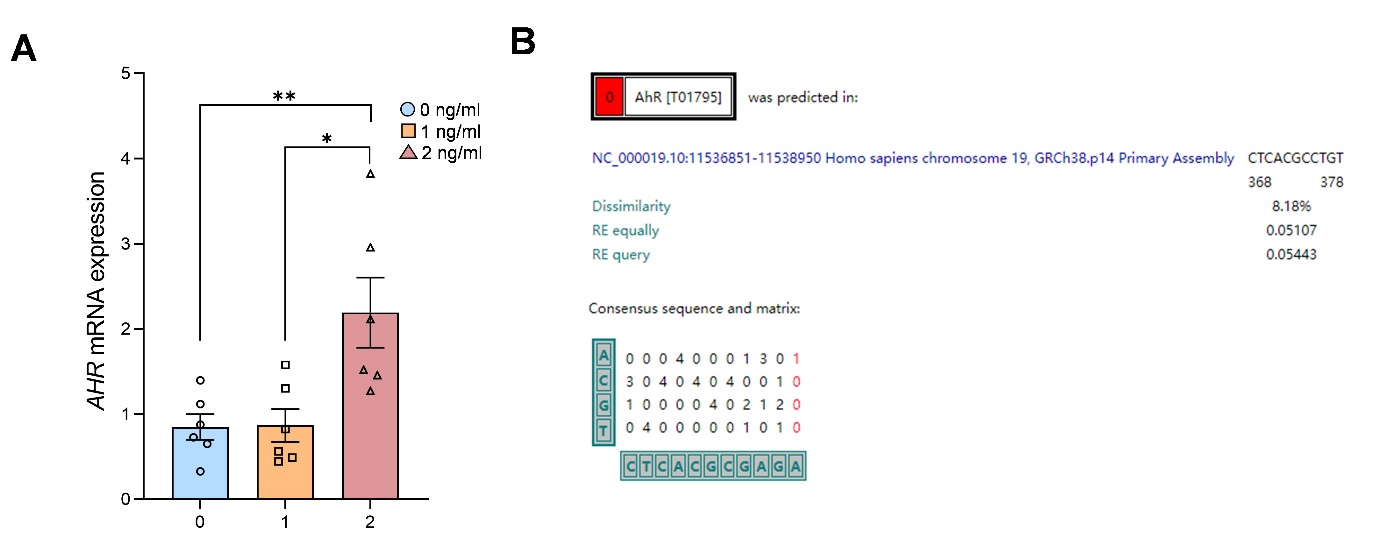
**

**Figure S10**. **Screening for the downstream target of skatole and potential transcriptional regulator of *CNN1*.** (A) *AHR* expression at mRNA level. (B) Prediction of the consensus sequence and binding matrix of AHR within the *CNN1*promoter, derived from PROMO 3.0. *P* values were determined by one-way ANOVA with Tukey’s test in A. Data were shown as mean ± SEM (n = 6 independent experiments). ^*^*P* < 0.05; ^**^*P* < 0.01.

**Table S1.** **Characteristics of study participants in the discovery cohort involved in study**

| **Characteristic** | **Total subjects** | **non−CAD** | **CAD** | ***P*** |
| --- | --- | --- | --- | --- |
|  | **(n=149)** | **(n=50)** | **(n=99)** |  |
| **Clinical characteristics** | |  |  |  |
| Male n (%) | 100 (67.1) | 26 (52.0) | 74 (74.8) | 0.009 |
| Age (years) | 62.00 [54.0, 68.0] | 60.50 [53.0, 68.8] | 63.00 [56.0, 68.0] | 0.257 |
| BMI (kg/m^2^) | 25.09 [22.8, 27.0] | 24.6 [22.5, 26.2] | 25.21 [22.8, 27.4] | 0.092 |
| **Disease history** | |  |  |  |
| Diabetes mellitus n (%) | 32 (21.5) | 9 (18.0) | 23 (23.2) | 0.601 |
| Hypertension n (%) | 93 (62.4) | 24 (48.0) | 69 (69.7) | 0.016 |
| Dyslipidemia n (%) | 35 (23.5) | 10 (20.0) | 25 (25.3) | 0.610 |
| Myocardial infarction n (%) | 11 (7.4) | 0 (0.0) | 11 (11.1) | 0.034 |
| **Baseline cardiovascular characteristics** | | |  |  |
| LMCAS n (%) | 18 (12.1) | 1 (2.0) | 17 (17.2) | 0.016 |
| LADS n (%) | 104 (69.8) | 21 (42.0) | 83 (83.8) | <0.001 |
| LCXS n (%) | 82 (55.0) | 8 (16.0) | 74 (74.8) | <0.001 |
| RCAS n (%) | 102 (68.5) | 20 (40.0) | 82 (82.8) | <0.001 |
| MVD n (%) | 49 (32.9) | 0 (0.0) | 49 (49.5) | <0.001 |
| Stents n (%) | 24 (16.1) | 0 (0.0) | 24 (24.2) | <0.001 |
| Median Gensini score | 12.0 [3.0,31.5] | 2.0 [0.0,4.9] | 22.0 [11.0,41.0] | <0.001 |
| SYNTAX score | 27.0 [21.4,32.8] | 24.5 [19.3,29.6] | 28.0 [23.4,33.4] | 0.015 |
| **Laboratory measurements** | |  |  |  |
| LVEF (%) | 67.0 [63.0,70.0] | 67.5 [65.0,71.8] | 66.0 [62.0,70.0] | 0.003 |
| SBP (mmHg) | 128.0 ±18.4 | 125.7 ±15.5 | 129.1 ±19.6 | 0.260 |
| DBP (mmHg) | 78.0 [72.0, 84.0] | 76.0 [71.0, 82.8] | 78.0 [72.5, 85.0] | 0.292 |
| TC (mmol/L) | 4.5 [3.9, 5.2] | 4.6 [3.9, 5.3] | 4.39 [3.7, 5.2] | 0.389 |
| HDL-c (mmol/L) | 1.1 [0.9, 1.2] | 1.2 [1.0, 1.3] | 1.0 [0.9, 1.2] | <0.001 |
| LDL-c (mmol/L) | 2.8 [2.3, 3.4] | 2.9 [2.4, 3.4] | 2.8 [2.3, 3.5] | 0.458 |
| TG (mmol/L) | 1.5 [1.1, 1.9] | 1.3 [1.0, 1.6] | 1.5 [1.1, 2.0] | 0.005 |
| ApoB (g/L) | 0.8 [0.7,1.0] | 0.8 [0.7,1.0] | 0.9 [0.7,1.0] | 0. 697 |
| NT-proBNP (pg/mL) | 71.6 [34.2, 181.5] | 49.4 [31.2, 116.3] | 80.3 [35.4, 222.3] | 0.038 |
| hs-TNT (pg/mL) | 7.7 [6.2, 11.3] | 6.3 [4.9, 9.1] | 8.8 [6.9, 13.0] | <0.001 |
| CK (U/L) | 96.3 [79.0,133.0] | 96.3 [86.1,111.1] | 96.0 [74.0,139.6] | 0.352 |
| CK-MB (U/L) | 13.7[12.0,15.4] | 13.0 [12.0,14.1] | 14.0 [12.0,16.0] | 0.008 |
| **Medication use** |  |  |  |  |
| Anti-diabetic drugs n (%) | 127 (85.2) | 43 (86.0) | 84 (84.9) | 0.999 |
| Anti-platelet n (%) | 121 (81.2) | 45 (90.0) | 76 (76.8) | 0.084 |
| Statin n (%) | 39 (26.2) | 5 (10.0) | 34 (34.3) | 0.003 |
| ACE inhibitor n (%) | 145 (97.3) | 50 (100.0) | 95 (96.0) | 0.366 |
| ARBs n (%) | 127 (85.2) | 45 (90.0) | 82 (82.8) | 0.357 |
| β-Blocker n (%) | 116 (77.9) | 43 (86.0) | 73 (73.7) | 0.135 |

CAD was defined as the presence of ≥ 50% coronary artery stenosis, whereas non-CAD was defined as < 50% stenosis. The following abbreviations were used: BMI indicates body mass index; LMCAS, left main coronary stenosis; LADS, left anterior descending artery stenosis; LCXS, left circumflex artery stenosis; RCAS, right coronary stenosis; MVD, multivessel coronary artery disease; LVEF, left ventricular ejection fraction; SBP, systolic blood pressure, DBP, diastolic blood pressure; TC, total cholesterol; HDL-c, high-density lipoprotein cholesterol and LDL-c, low-density lipoprotein cholesterol; TG, total triglycerides; NT-proBNP, N-terminal prohormone of brain natriuretic peptide. Data were shown as mean ± SD or median (interquartile range), and *P* values were determined by Student’s *t*-test or Wilcoxon rank-sum test.

**Table S2. Comparison of basic characteristics of the study participants in discovery and validation cohort**

| **Characteristic** | **Total subjects** | **Discovery cohort** | **Validation Cohort** | ***P*** |
| --- | --- | --- | --- | --- |
|  | **(n=328)** | **(n=149)** | **(n=179)** |  |
| **Clinical characteristics** |  |  |  |  |
| Male n (%) | 201 (61.3) | 100 (67.1) | 101 (56.4) | 0.062 |
| Age (years) | 62.0 [54.0, 67.0] | 62.0 [54.0, 68.00] | 61.0 [54.0, 67.0] | 0.181 |
| BMI (kg/m^2^) | 25.1 [22.7, 26.9] | 25.1 [22.8, 27.0] | 25.1 [22.7, 26.7] | 0.833 |
| **Disease history** |  |  |  |  |
| Diabetes mellitus n (%) | 69 (21.0) | 32 (21.5) | 37 (20.7) | 0.966 |
| Hypertension n (%) | 192 (58.5) | 93 (62.4) | 99 (55.3) | 0.235 |
| Dyslipidemia n (%) | 77 (23.5) | 35 (23.5) | 42 (23.5) | 0.999 |
| Myocardial infarction n (%) | 26 (7.9) | 11 (7.4) | 11 (11.1) | 0.898 |
| **Baseline cardiovascular characteristics** | |  |  |  |
| LMCAS n (%) | 33 (10.1) | 18 (12.1) | 15 (8.4) | 0.355 |
| LADS n (%) | 205 (62.5) | 104 (69.8) | 101 (56.4) | 0.017 |
| LCXS n (%) | 147 (44.8) | 82 (55.0) | 65 (36.3) | 0.001 |
| RCAS n (%) | 197 (60.1) | 102 (68.5) | 95 (53.1) | 0.007 |
| MVD n (%) | 79 (24.1) | 49 (32.9) | 30 (16.8) | 0.001 |
| Stents n (%) | 24 (7.3) | 24 (16.1) | 0 (0.0) | <0.001 |
| Median Gensini score | 10.6 [4.0, 26.3] | 12.0 [3.0,31.5] | 9.6 [4.8, 24.2] | 0.051 |
| SYNTAX score | 27.5 [23.8, 31.6] | 27.2 [22.1, 32.2] | 27.6 [24.5, 31.0] | 0.213 |

BMI: body mass index; LMCAS: left main coronary stenosis; LADS: left anterior descending artery stenosis; LCXS: left circumflex artery stenosis; RCAS: right coronary stenosis; MVD: multivessel coronary artery disease. Data were expressed as mean ± SD or median (interquartile range), and *P* values were determined by Student’s *t*-test or Wilcoxon rank-sum test.

**Table S3. Primers used in this study**

| **Species** | **Primer** | **Sequence (5’→3’)** |  |
| --- | --- | --- | --- |
| **Mouse** | *SMA* | F: ACTACTGCCGAGCGTGAGAT | R: AGGTAGACAGCGAAGCCA |
|  | *Myh11* | F: CTCAAGAGCAAACTCAGAGG | R: ACACCCTTTGTGCAGGGCTGA |
|  | *Tagln* | F: CAACAAGGGTCCATCCTACGG | R: ATCTGGGCGGCCTACATCA |
|  | *Cnn1* | F: GAAGGTCAATGAGTCAACTCAGAA | R: CCATACTTGGTAATGGCTTTGA |
|  | *Ki67* | F: CCTGCCTCAGATGGCTCAAA | R: GGTTCCCTGTAACTGCTCCC |
|  | *β-actin* | F: AGAAGATCTGGCACCACACC | R: TACGACCAGAGGCATACAGG |
| **Human** | *SMA* | F: CGCTGTCAGGAACCCTGAGA | R: CGAAGCCGGCCTTACAGA |
|  | *MYH11* | F: TGAGCCGAGATTGCACCAA | R: TGCCACAGGGGATATAAGCC |
|  | *TAGLN* | F: AGTGCAGTCCAAAATCGAGAAG | R: CTTGCTCAGAATCACGCCAT |
|  | *CNN1* | F: CTGTCAGCCGAGGTTAAGAAC | R: GAGGCCGTCCATGAAGTTGTT |
|  | *Ki67* | F: GAATTGAACCTGCGGAAGAGC | R: AGCGCAGGGATATTCCCTTATTTT |
|  | *β-actin* | F: CGTCACCAACTGGGACGACA | R: CTTCTCGCGGTTGGCCTTGG |
|  | *CNN1promoter* for ChIP | F: GTCAACCCAAAATTGGCACCA | R: ACCTTGTTTCCTTTCGTCTTCG |
| **Bacteria** | *Olsenella scatoligenes* | F: CTTACCAGGGCTTGACATCTTGG | R: ACGACACGAGCTGACGACAG |
|  | IAD | F: GGCTCCTATCGCCTACACCA | R: ATCGGCTTGACCTCCTCCTC |
|  | Total bacteria | F: ACTCCTACGGGAGGCAGCAG | R: ATTACCGCGGCTGCTGG |

**Table S4. Summary of animal study design**

| **Groups** | **Sex** | **Age** | **Number (prior to experiment)** | **Number (after termination)** | **Littermates**  **(Yes/No)** | **Other description** |
| --- | --- | --- | --- | --- | --- | --- |
| Group 1  (Vehicle control) | Male | 8 weeks | 8 | 8 | Yes | N/A |
| Group 2  (*O. scatoligenes* intervention) | Male | 8 weeks | 8 | 8 | Yes | N/A |
| Group 3  (shCon+CMC-Na intervention) | Male | 8 weeks | 8 | 8 | Yes | N/A |
| Group 4  (shCon+ Skatole intervention) | Male | 8 weeks | 8 | 8 | Yes | N/A |
| Group 5  (shCnn1+ CMC-Na intervention) | Male | 8 weeks | 8 | 8 | Yes | N/A |
| Group 6  (shCnn1+ Skatole intervention) | Male | 8 weeks | 8 | 8 | Yes | N/A |

**Table S5. Summary of statistical analyses**

| **Analysis Category** | **Specific Analysis** | **Statistical Test / Method** | **Software / Package** | **Significance Threshold** | **Corrections / Adjustments** |
| --- | --- | --- | --- | --- | --- |
| Data Preprocessing | Data Preprocessing | Quality Control, Taxonomic Profiling | In-house scripts, MetaPhlAn 3.0, HUMAnN3 | - | - |
| Alpha Diversity | Shannon Index | Wilcoxon rank-sum test | vegan (R) | *P* < 0.05 | - |
| Beta Diversity | Bray-Curtis Dissimilarity, dbRDA, PERMANOVA | Permutational Multivariate ANOVA (adonis) | vegan (R) | *P* < 0.05 | - |
| Differential Abundance (Microbes, Functions, Metabolites) | Species, KOs, Metabolites | Linear Models (MaAsLin2) | MaAsLin2 (R) | FDR-adjusted *P* < 0.2 | Adjusted for sex, age, BMI, medication |
| Co-abundance Network | Network Construction | Spearman Correlation | ggraph, igraph (R) | Permuted adjusted *P* < 0.2, \|rho\| > 0.2 | - |
| Network Properties | Degree, Betweenness, Closeness | Student's t-test | igraph (R) | FDR-adjusted *P* < 0.05 | - |
| Clinical Correlations | O. scatoligenes vs. Parameters | Spearman / Partial Spearman Correlation | Base R | Permuted adjusted *P* < 0.2 | Adjusted for sex, age, BMI, medication (partial) |
| RNA-Seq Analysis | Differential Gene Expression | DESeq2 | DESeq2, tximport (R) | FDR-adjusted *P* < 0.2, \|log2FC\| > 1 | - |
| GO Enrichment | Pathway Enrichment | Hypergeometric Test | clusterProfiler (R) | FDR-adjusted *P* < 0.05 | - |
| Gene Correlation Network | VSMC Proliferation Genes | Spearman Correlation | igraph (R) | FDR-adjusted *P* < 0.1 | - |

**References**

1. Ibanez B, James S, Agewall S *et al.* 2017 esc guidelines for the management of acute myocardial infarction in patients presenting with st-segment elevation: The task force for the management of acute myocardial infarction in patients presenting with st-segment elevation of the european society of cardiology (esc). *Eur. Heart J.* 2018;**39**:119-177 <https://doi.org/10.1093/eurheartj/ehx393>

2. Gensini GG. A more meaningful scoring system for determining the severity of coronary heart disease. *Am. J. Cardiol.* 1983;**51**:606 <https://doi.org/10.1016/s0002-9149(83)80105-2>

3. Levine GN, Bates ER, Blankenship JC *et al.* 2011 accf/aha/scai guideline for percutaneous coronary intervention: A report of the american college of cardiology foundation/american heart association task force on practice guidelines and the society for cardiovascular angiography and interventions. *Circulation*. 2011;**124**:e574-651 <https://doi.org/10.1161/CIR.0b013e31823ba622>

4. 2. Classification and diagnosis of diabetes: Standards of medical care in diabetes-2019. *Diabetes Care*. 2019;**42**:S13-s28 <https://doi.org/10.2337/dc19-S002>

5. Whelton PK, Carey RM, Aronow WS *et al.* 2017 acc/aha/aapa/abc/acpm/ags/apha/ash/aspc/nma/pcna guideline for the prevention, detection, evaluation, and management of high blood pressure in adults: A report of the american college of cardiology/american heart association task force on clinical practice guidelines. *J. Am. Coll. Cardiol.* 2018;**71**:e127-e248 <https://doi.org/10.1016/j.jacc.2017.11.006>

6. Grundy SM, Stone NJ, Bailey AL *et al.* 2018 aha/acc/aacvpr/aapa/abc/acpm/ada/ags/apha/aspc/nla/pcna guideline on the management of blood cholesterol: A report of the american college of cardiology/american heart association task force on clinical practice guidelines. *Circulation*. 2019;**139**:e1082-e1143 <https://doi.org/10.1161/cir.0000000000000625>

7. Chen W, Gong L, Guo Z *et al.* A novel integrated method for large-scale detection, identification, and quantification of widely targeted metabolites: Application in the study of rice metabolomics. *Mol Plant*. 2013;**6**:1769-1780 <https://doi.org/10.1093/mp/sst080>

8. Luo S, Zhao Y, Zhu S *et al.* *Flavonifractor plautii* protects against elevated arterial stiffness. *Circ. Res.* 2023;**132**:167-181 <https://doi.org/10.1161/circresaha.122.321975>

9. Li J, Sung CY, Lee N *et al.* Probiotics modulated gut microbiota suppresses hepatocellular carcinoma growth in mice. *Proc. Natl. Acad. Sci. U. S. A.* 2016;**113**:E1306-1315 <https://doi.org/10.1073/pnas.1518189113>

10. Beghini F, McIver LJ, Blanco-Míguez A *et al.* Integrating taxonomic, functional, and strain-level profiling of diverse microbial communities with biobakery 3. *Elife*. 2021;**10**:e65088 <https://doi.org/10.7554/eLife.65088>

11. Oksanen J, Kindt R, Legendre P *et al.* *The vegan package. Vegan: Community ecology package*, 2010.

12. Ni Y, Qian L, Siliceo SL *et al.* Resistant starch decreases intrahepatic triglycerides in patients with nafld via gut microbiome alterations. *Cell Metab.* 2023;**35**:1530-1547.e1538 <https://doi.org/10.1016/j.cmet.2023.08.002>

13. Patro R, Duggal G, Love MI *et al.* Salmon provides fast and bias-aware quantification of transcript expression. *Nat Methods*. 2017;**14**:417-419 <https://doi.org/10.1038/nmeth.4197>

14. Soneson C, Love MI, Robinson MD. Differential analyses for rna-seq: Transcript-level estimates improve gene-level inferences. *F1000Res*. 2015;**4**:1521 <https://doi.org/10.12688/f1000research.7563.2>
